# With or without a Ca^2+^ signal?: a proteomics approach toward Ca^2+^-dependent and -independent changes in response to oxidative stress in *Arabidopsis thaliana*

**DOI:** 10.1007/s00425-025-04891-y

**Published:** 2025-12-05

**Authors:** Annelotte van Dieren, Andras Bittner, Bernhard Wurzinger, Leila Afjehi-Sadat, Wolfram Weckwerth, Markus Teige, Ute C. Vothknecht

**Affiliations:** 1 Institute for Cellular and Molecular Botany, University of Bonn, Kirschallee 1, 53115 Bonn, Germany; 2 Molecular Systems Biology (MOSYS), Department of Functional and Evolutionary Ecology, University of Vienna, Djerassiplatz 1, 1030 Vienna, Austria; 3 Mass Spectrometry Unit, Research Support Facilities, University of Vienna, Djerassiplatz 1, 1030 Vienna, Austria; 4 Institute of Plant Biotechnology and Cell Biology, Department of Biotechnology and Food Sciences, BOKU University, Vienna, Austria; 5 Vienna Metabolomics Center (VIME), University of Vienna, Djerassiplatz 1, 1030 Vienna, Austria

**Keywords:** Calcium signaling, ROS, H_2_O_2_, Abiotic stress, Biotic stress, Cross-talk

## Abstract

**Main conclusion:**

Our work identified Ca^2+^-dependent and -independent changes in protein contents upon oxidative stress, showing that Ca^2+^ signaling shapes the early oxidative stress response and identifying potential targets for stress resilience research.

**Abstract:**

Calcium (Ca^2+^) and reactive oxygen species (ROS) are key secondary messengers in plant stress signaling, yet their interplay in regulating proteome-wide responses remains poorly understood. We employed label-free quantitative (LFQ) proteomics to investigate Ca^2+^-dependent and -independent proteome changes in *Arabidopsis thaliana* leaves upon oxidative stress induced by hydrogen peroxide (H_2_O_2_). To dissect the role of Ca^2+^ signaling, we inhibited H_2_O_2_-induced Ca^2+^ transients by pre-treatment with the Ca^2+^ influx blocker LaCl_3_. Throughout all four treatment samples - control, H_2_O_2_-treated, LaCl_3_-treated, H_2_O_2_- and LaCl_3_-treated - we identified a total of 3724 and 3757 proteins after 10 and 30 min, respectively. Of these, 581 proteins showed significant changes in abundance between the 10 min and 909 proteins between the 30 min sample groups. The combined LaCl_3_ and H_2_O_2_ treatment resulted in the highest number of differentially abundant proteins (DAPs), indicating a strong attenuating effect of Ca^2+^ signaling on the oxidative stress response. By contrast, only 37 and 57 proteins responded to H_2_O_2_ alone with distinct subsets of strictly Ca^2+^-dependent, partially Ca^2+^-dependent, and Ca^2+^-independent proteins. Ca^2+^-independent H_2_O_2_-responsive proteins predominantly showed increased abundance, while strictly Ca^2+^-dependent proteins exhibited decreased abundance, suggesting a role for Ca^2+^ signaling in protein degradation. Furthermore, three proteins—WLIM1, CYP97C1, and AGAP1—underwent shifts in Ca^2+^-dependency between the two time points, pointing to a dynamic Ca^2+^-regulation. This study provides insight into short-term Ca^2+^-dependent and independent regulation of the *Arabidopsis* leaf proteome in response to oxidative stress, thereby identifying potential new targets for research on plant stress resilience mechanisms.

**Supplementary Information:**

The online version contains supplementary material available at 10.1007/s00425-025-04891-y.

## Introduction

Plants are continuously exposed to various environmental stresses, including drought, salinity, extreme temperatures, and pathogen attacks. These stressors can disrupt cellular homeostasis, leading to the overproduction of reactive oxygen species (ROS), which can cause oxidative damage to cellular components, such as lipids, proteins, and nucleic acids (Mittler 2017). However, plants have evolved sophisticated signaling networks to perceive and respond to oxidative stress, with calcium signaling playing a central role in orchestrating these adaptive responses (Li et al. 2022). Calcium ions (Ca^2+^) serve as a ubiquitous second messenger in plant cells, regulating various physiological and developmental processes. A diverse array of biotic and abiotic stress factors, along with various developmental processes, can induce transient increases in cytosolic free Ca^2+^ concentration ([Ca^2+^]_cyt_) through a regulated influx of Ca^2+^ from both extracellular sources and intracellular reservoirs into the cytosol (McAinsh and Pittman 2009; Kudla et al. 2010). These transient elevations in [Ca^2+^]_cyt_ exhibit distinct spatio-temporal characteristics, including variations in amplitude, frequency, and subcellular localization, in a manner that is specific to the type of stimulus encountered. The unique patterns of [Ca^2+^]_cyt_ fluctuations, commonly referred to as “calcium signatures” (Allen et al. 2001; Whalley and Knight 2013) play a crucial role in ensuring the specificity of calcium-mediated signaling, thereby facilitating context-dependent and stimulus-appropriate cellular responses. Each calcium signature arises from the coordinated and dynamic interplay of multiple Ca^2+^ influx channels and efflux transporters, which are located within the plasma membrane as well as the membranes of various intracellular organelles (Demidchik et al. 2018). They are decoded by Ca^2+^-binding proteins, such as calmodulins (CaMs) and calmodulin-like proteins (CMLs), calcium-dependent protein kinases (CDPKs), and calcineurin B-like proteins (CBLs), as well as C2-domain-containing proteins which together transduce the signal to downstream effectors (Pappan et al. 2004; Mohanta et al. 2019; Tang et al. 2020). Furthermore, different plant organs and tissues exhibit distinct calcium signatures in response to stress, emphasizing the complexity and specificity of Ca^2+^-mediated signaling networks (Costa et al. 2018; Giridhar et al. 2022).

After the initial identification of stimulus-induced changes in [Ca^2+^]_cyt_, an increasing number of processes involving Ca^2+^ signaling have been elucidated, including those related to plant growth and development, such as cell division and organ formation (Zhang et al. 2014). Numerous studies have investigated Ca^2+^ transient-inducing stimuli, such as NaCl, Mannitol, flg22, chitin in *Arabidopsis* (Knight et al. 1997; Yuan et al. 2020) and across different crop species, including NaCl, mannitol, H_2_O_2_, and Flg22 in barley leaf and root samples (Giridhar et al. 2022), and NaCl, mannitol, H_2_O_2_ and Pep13 in potato (van Dieren et al. 2024). Additionally, downstream responses controlled by these specific Ca^2+^ signals have been described, including the role of Ca^2+^-regulated kinases in mediating phosphorylation events that coordinate signaling cascades (Ludwig 2003) as well as responses that comprise regulation of gene expression through Ca^2+^-regulated transcriptional responses (Kaplan et al. 2006) and Ca^2+^-responsive promotor elements (Kudla et al. 2010).

Oxidative stress results from an imbalance between ROS production and detoxification. While excessive ROS can be detrimental, controlled ROS production acts as a secondary messenger that activates stress-responsive pathways (Mittler 2017; Chen and Yang 2020). Rapid signaling and communication from individual cells that perceive potential threats to their neighboring cells as well as more distal tissue is vital for plant acclimation and fitness. In this context, it was shown that Ca^2+^ signaling and ROS can interact in complex feedback loops. ROS can induce Ca^2+^ influx through plasma membrane and organellar channels, leading to further signal propagation (Li et al. 2022; Ravi et al. 2023). In turn, Ca^2+^ signaling modulates ROS-scavenging mechanisms, such as the activation of antioxidant enzymes including superoxide dismutase (SOD), catalase (CAT), and ascorbate peroxidase (APX) (Gilroy et al. 2016). Other studies highlight the role of NADPH oxidases, also known as respiratory burst oxidase homologs (RBOHs), in ROS production upon Ca^2+^ signaling activation (Kärkönen and Kuchitsu 2015). These enzymes facilitate ROS bursts that act as secondary messengers, amplifying stress responses. Moreover, Ca^2+^ channels, such as cyclic nucleotide-gated channels (CNGCs) and glutamate receptor-like channels (GLRs), contribute to ROS–Ca^2+^ cross-talk, further fine-tuning the stress response (Gilroy et al. 2016).

One of the first layers of cellular responses to stimuli-induced signaling is the re-adjustments of the transcriptional activity. Consequently, many Omics-based studies asses stress responses by quantifying changes in gene expression. However, proteins are key players in the structure, function, and regulation of cells, tissues, and organs, and proteome changes can occur independent from transcription by processes such as protein degradation and regulation of translation (Gry et al. 2009; Payne 2015; Liu et al. 2016). The interplay of ROS and Ca^2+^ signaling on transcriptome changes have recently been investigated in barley (Bhattacharyya et al. 2025), however, no investigation has so far described the effect of Ca^2+^ signaling on ROS induced changes of proteomes. In this work, we thus aimed to elucidate the role of H_2_O_2_-induced Ca^2+^ signals on short-term proteome changes observed in *Arabidopsis* leaf tissue by inhibiting stress-induced Ca^2+^ transients via LaCl_3_, a widely used extracellular blocker of Ca^2+^ influx (Rentel and Knight 2004; Tracy et al. 2008). MS-based proteome analysis identified specific subsets of proteins, whose abundance changed after 10 and 30 min of H_2_O_2_ application in a Ca^2+^-dependent or -independent manner. The integration of these proteomic data with other omics datasets (e.g., genomics, transcriptomics, and metabolomics) can advance our understanding of complex regulatory processes. While obtained from Arabidopsis, this knowledge could be applied to future research aiming to enhance stress resistance and optimizing performance and productivity in crop species under increasingly challenging environmental conditions.

## Materials and methods

### Plant material and growth conditions

Leaf protein extracts for the proteomics analysis were obtained from *A. thaliana* (ecotype Columbia; Col-0). Seeds were sown on soil, stratified for 2 days at 4 °C in the dark, and separated after germination into single pots filled with standard plant potting soil pre-treated with Confidor WG 70 (Bayer Agrar, Germany). Plants were cultivated in a climatized growth chamber with a room temperature of 20 ± 2 °C, a light intensity of ~ 150 µmol photons m^−2^ s^−1^ (Philips TLD 18 W of alternating 830/840 light color) and long day conditions (16 h light/8 h dark). Pre-experiments to determine the best conditions for the inhibitor treatment and stress stimulus were performed with Arabidopsis plants expressing cytosolic apoaequorin (At-AEQ_cyt_) (Knight et al. 1991).

### Aequorin reconstitution, luminescence measurements, and Ca^2+^ concentration calculations

Stimulus-induced Ca^2+^ transients were analyzed using leaf material of three-week-old At-AEQ_cyt_ plants. On the day before measurements were taken, leaf disks (Ø 6 mm) were collected and incubated overnight in the dark at 20°C in 5 µM coelenterazine (Biosynth AG, Switzerland) for reconstitution of the cytosol targeted apoaequorin to aequorin. After reconstitution, leaf disks were carefully washed (ddH_2_O) and transferred either into 1 mM LaCl_3_ solution (inhibitor pre-treatment) or ddH_2_O (mock) for 1 h. Subsequently, single leaf disks were washed again and transferred individually into a 96-well plate (Lumitrac 600, Greiner Bio-One, Austria), floating in 100 µl ddH_2_O. Photon count measurements were performed using a plate luminometer (Tristar 2 Multimode Reader, Berthold GmbH). First, the basal level of photon counts was measured for 30 s with an interval of 1 s, followed by the application 20 mM H_2_O_2_ using a 40 mM stock solution and a volume equal to the starting volume of the ddH_2_O (100 µl), with continuous measuring of the response for 240 s. The remaining aequorin was discharged by adding discharge solution (final concentration of 1 M CaCl_2_ in 10% (v/v) EtOH) and photon counts were recorded for another 300 s. Concentrations of free calcium ions in the cytosol ([Ca^2+^]_cyt_) were calculated based on the photon counts as described before (Knight and Knight 1995). The measurements were performed with three independent experimental replicates consisting of three technical replicates.

### Sample collection and treatment for proteomics

**Fig. 1:**
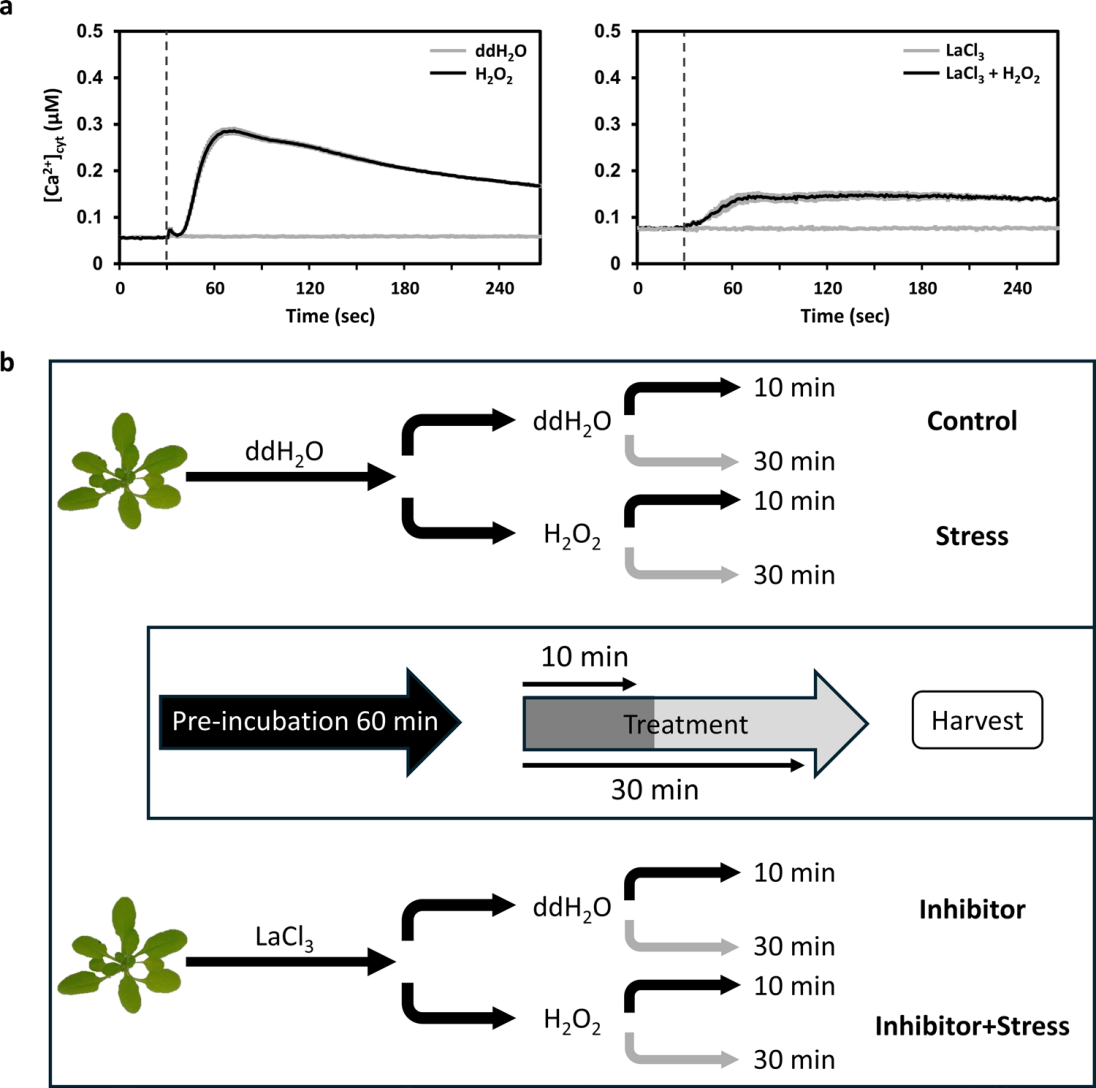
Experimental background and design. **a** Time course of changes in [Ca^2+^]_cyt_ in response to 20 mM H_2_O_2_ in leaf tissue of Arabidopsis (left) and in response to 20 mM H_2_O_2_ after 60 min pre-incubation with LaCl_3_ (right). Values are shown as mean ± SE (*n* = 6). Dashed vertical lines indicate the time point of stimuli injection (30 s). **b** Overview of treatment application: plants were either pre-incubated in ddH_2_O or 1 mM LaCl_3_ for 60 min. Half of the plants from both pre-incubations were transferred into a 20 mM H_2_O_2_ solution, another half was transferred into fresh ddH_2_O. Half of these plants were harvested after 10 min, the other half after 30 min and labelled as indicated: Control (ddH_2_O + ddH_2_O), Stress (ddH_2_O + 20 mM H_2_O_2_), Inhibitor (1 mM LaCl_3_ + ddH_2_O), Inhibitor + Stress (1 mM LaCl_3_ + 20 mM H_2_O_2_) with their corresponding treatment duration (10 or 30 min)

For proteomics analysis, complete rosettes of three-weeks-old Col-0 plants were incubated in 1 mM LaCl_3_ (inhibitor treatment) or ddH_2_O for 1 h. ddH_2_O and LaCl_3_ pre-treated plants were carefully washed and then transferred into either 20 mM H_2_O_2_ (stress treatment) or ddH_2_O for the control treatment. For each proteomics sample, complete rosettes of 12 plants were harvested after 10 and 30 min of the stress treatment, pooled, immediately frozen in liquid nitrogen, and stored at -80 °C until protein extraction. Within one experiment, plants from all four treatments were harvested for both timepoints (10 and 30 min), and a total of 5 experiments with independently grown plants were performed. For a schematic overview of the protocol see Fig. 1.

### Protein isolation, precipitation, lysis, and digestion

Frozen plant material was first ground in liquid nitrogen using a pre-cooled mortar and pestle. 500 mg of the ground plant material was mixed with 2 ml ice-cold Lacus protein isolation buffer (20 mM Tris-HCl [pH 7.7], 80 mM NaCl, 0.75 mM EDTA, 1 mM CaCl_2_, 5 mM MgCl_2_, 1 mM DTT, 1 mM NaF) containing 4 tablets of protease inhibitor (Roche cOmplete EDTA-free, protease inhibitor cocktail tablets) and 10 tablets of phosphatase inhibitor (Roche PhosSTOP) per 200 ml. Samples were incubated on ice for 10 min followed by centrifugation at 15,000*g* for 10 min at 4 °C. Supernatants were transferred into a fresh tube. An equal volume of 20% (w/v) trichloroacetic acid (TCA) was added to the supernatant, and the samples were placed on ice for 30 min. Afterward, the samples were centrifuged at 15,000*g* for 10 min at 4 °C and the supernatant removed. The precipitated protein pellets were washed with cold 80% acetone and the samples were vacuum-dried. 50 µl of urea lysis buffer (8 M urea, 150 mM NaCl, 40 mM Tris–HCl pH 8) was added and the protein concentration was determined via the Pierce™ BCA Protein Assay (Thermo Fisher Scientific). Subsequently, 3 mg total protein per sample was reduced in 5 mM DTT and alkylated in 15 mM iodoacetamide for 30 min in the dark at room temperature. The alkylated samples were quenched by adding DTT to a final concentration of 5 mM and mixed with 30 mg carboxylate beads (Sera-Mag™, 1:1 ratio of hydrophilic and hydrophobic beads, Cytiva, USA). Proteins attached to the beads were washed four times with 80% (v/v) ethanol and digested in ammonium bicarbonate buffer (30 mM, pH 8.2) containing 30 µg trypsin (Promega, WI, USA). Tryptic digestion was performed overnight at 37 °C under constant shaking. The digestion was stopped by the addition of formic acid (final concentration 4%). 100 µg of the digested peptides per sample were transferred into a new reaction tube, vacuum-dried and stored at -20 °C until HPLC–MS analysis.

### LC–MS analysis

Digested peptides were subjected to LC–MS analysis at the mass spectrometry unit of the faculty of life sciences at the University of Vienna as described previously (Bleker et al. 2024). In brief: approximately 1 µg of peptide sample was reconstituted in 0.1% (v/v) formic acid and separated using an online reversed-phase high-pressure liquid chromatography (HPLC) system. Separation was performed on a heated C18 analytical column over a 140-min gradient (5–50%). The eluate was introduced into a Q-Exactive Plus mass spectrometer using an Easy-Spray ion source. Mass spectra were acquired in positive ion mode with a data-dependent acquisition strategy, selecting the top 15 most intense ions for MS analysis. A full MS scan was performed at 70,000 resolution (*m*/*z* 200), followed by MS/MS fragmentation at 17,500 resolution using higher-energy collisional dissociation (HCD) at 27% normalized collision energy. Dynamic exclusion was set to 40 s, and specific precursor ions (unassigned, + 1, + 7, + 8, and > + 8 charge states) were excluded. The analysis was conducted with five independent experimental replicates per sample to ensure reproducibility.

### Peptide identification and quantification

Identities and peptide features were defined by the peptide search engine Andromeda provided by the MaxQuant software (Prianichnikov et al. 2020) and using standard settings (Tyanova et al. 2016a, b). In detail: trypsin-based digestion of the peptides with up to two missing cleavage sites was selected. Methionine oxidation as well as N-terminal acetylation was set as a variable modification for peptide identification. In total, up to three potential modification sites per peptide were accepted. The identified peptide sequences were searched and aligned against the Araport11 reference protein database (Cheng et al. 2017). The false-discovery rate cut-off for protein identification and side identification was set to 0.01. The minimum peptide length was set to seven and the maximum length to 40 amino acids. On average, proteins for the 10 min dataset were identified by 12.3 peptides, of which 9.9 were unique, with a mean sequence coverage of 31.5% (25.7% when considering only unique peptides). Similarly, proteins in the 30 min dataset were identified by an average of 12.2 peptides, of which 9.8 were unique, with a mean sequence coverage of 31.8% (25.8% for unique peptides). For each identified protein group, label-free quantitation (LFQ) intensities were calculated using the Maxquant software. A protein group contains all proteins and protein isoforms that cannot be unambiguously identified by unique peptides but have shared peptides. We further on refer to protein groups only as ‘proteins’.

### Data analysis

For quantitative proteome analysis, the derived LFQ intensities were loaded into the Perseus software (Tyanova et al. 2016a, b) and used for data and statistical analysis, as well as graphics and visualization of the results. The steps taken to determine which proteins show a differential abundance among the different treatments was based on a method described before (Nikonorova et al. 2018). In short: after loading the LFQ intensities, a quality control was performed in which protein groups with the indication ‘only identified by site’ (proteins that are only identified by peptides carrying modified amino acids), reverse sequences (decoy proteins), and potential contaminants (for example albumin) were filtered out. Biological replicates were grouped, and values were *log2* transformed. Protein groups were filtered based on valid values, where the criterion was set to have at least 3 valid values in one group (each treatment group consists of 5 replicates) to remove the low abundant proteins. Remaining missing values (protein group not identified in a run) were imputed with values based on the normal distribution with a width of 0.3 (relative to the standard deviation of the measured values) and a downshift of 1.8. The imputed numbers represent very small values, meaning the identified peptide has a very low abundance. Principal component analysis (PCA) was performed using the Perseus software. Differential abundant proteins were determined by multiple sample ANOVA test (*p* value < 0.05). *p* values were corrected for multiple testing using Benjamin–Hochberg rule (adjusted *p* value). All ANOVA significant proteins were z score-normalized and used for supervised hierarchical clustering to produce a heatmap, using Euclidean distance and average linkage. Pairwise Student’s t tests (*p* value < 0.05) were performed on the non-z-scored values to determine differences in protein abundance between two treatments. Volcano plots were generated using the Perseus software, by plotting log_2_ fold change values on the *x*-axis against the − log *p* values on the *y*-axis, cut-off was set by nonlinear volcano lines based on S0 = 0.1 adjusted *p* value. Protein groups showing different abundance among the treatments were analyzed for overlapping groups between treatments and time points. Gene ontology (GO) enrichment analysis (KEGG, biological processes, molecular function) was performed on the major protein clusters identified by hierarchical clustering, and on the identified H_2_O_2_-responsive proteins using ShinyGO, which uses the annotations of Ensembl and STRING-db (Ge et al. 2020). An error probability according to Fisher’s *t* test of < 0.05 and a false-discovery rate (FDR) of < 0.01 was selected for enriched GO terms.

### Immunodetection

For immunodetection, proteins were isolated from the plant material that was used for the MS analysis using the same Lacus protein isolation buffer and isolation protocol (described above). The proteins were separated on a 12% SDS–polyacrylamide gel and transferred to nitrocellulose membrane (0.45µm pore size; Bio-Rad Laboratories). After transfer of the proteins, the membrane was stained using 0.1% (w/v) Ponceau S in 5% (v/v) glacial acetic acid. Immunodetection was performed using antibodies against Anti-PAL 1-4 (dilution 1:2000) and Anti-TIP 1;1 1;2 (dilution 1:1000) (Agrisera, AS21 4614 and AS22 4844). Blots were incubated with the matching secondary antibody (anti-rabbit IgG horse radish peroxidase conjugated, AS09602, dilution 1:25,000) and developed with the AgriseraBright (AS16 ECL-N-10) detection reagent.

### Statistical analysis

Proteomic data were analyzed using the Perseus software package (Tyanova et al. 2016a, b) for principal component analysis (PCA), hierarchical clustering, and volcano plot generation. Functional enrichment analysis of KEGG pathways and gene ontology (GO) terms were performed using ShinyGO, which calculated false-discovery rates (FDR) and fold enrichments.

## Results

H_2_O_2_ and Ca^2+^ are secondary messengers that are involved in the mediation of environmental changes into an appropriate cellular response. Temporal increases in these messengers affect various cellular processes including gene transcription or protein activity. Here we employed label-free quantitative (LFQ) proteomics to analyze the H_2_O_2_-induced changes in the leaf proteome of *Arabidopsis* and the contribution of Ca^2+^ signals in the H_2_O_2_-induced changes.

### Establishing parameters and experimental design

While shown before for *Arabidopsis* (Giridhar et al. 2022; Rentel and Knight 2004; van Dieren et al. 2024), we confirmed the H_2_O_2_ induced Ca^2+^ transients and their inhibition by LaCl_3_ under the experimental conditions chosen for the protein isolation. Leaf disks from 3-week-old *Arabidopsis* plants expressing cytosolic apoaequorin (At-AEQ_cyt_), grown under the same circumstances as the wild type plants, were incubated with coelenterazine to reconstitute aequorin. After pre-treatment with either ddH_2_O or 1 mM LaCl_3_, oxidative stress was mimicked by adding 20 mM H_2_O_2_ and changes in [Ca^2+^]_cyt_ were measured using a luminometer. As shown before for soil-grown *Arabidopsis* plants (van Dieren et al. 2024), the oxidative stress stimulus resulted in a well-shaped Ca^2+^ transient, which was inhibited by over 50% upon pre-treatment with LaCl_3_ (Fig. 1a).

The workflow of the application of the different treatments before proteomics analysis is schematically displayed in Fig. 1b. Complete rosettes from 3-week-old wild type plants grown on soil were pre-incubated with LaCl_3_ (Inhibitor) or ddH_2_O for 60 min. Subsequently, the rosettes were washed carefully and treated with either 20 mM H_2_O_2_ (Stress) or ddH_2_O for 10 and 30 min. The timing was chosen to elucidate short-term responses to the stress stimulus. The different treatment paths result in the following treatment names used further: Control: pre-incubation in ddH_2_O, treatment with ddH_2_O; Stress: pre-incubation with ddH_2_O, treatment with H_2_O_2_; Inhibitor: pre-incubation with LaCl_3_, treatment with ddH_2_O; Inhibitor + Stress: pre-incubation with LaCl_3_, treatment with H_2_O_2_.

### Initial data analysis

**Fig. 2:**
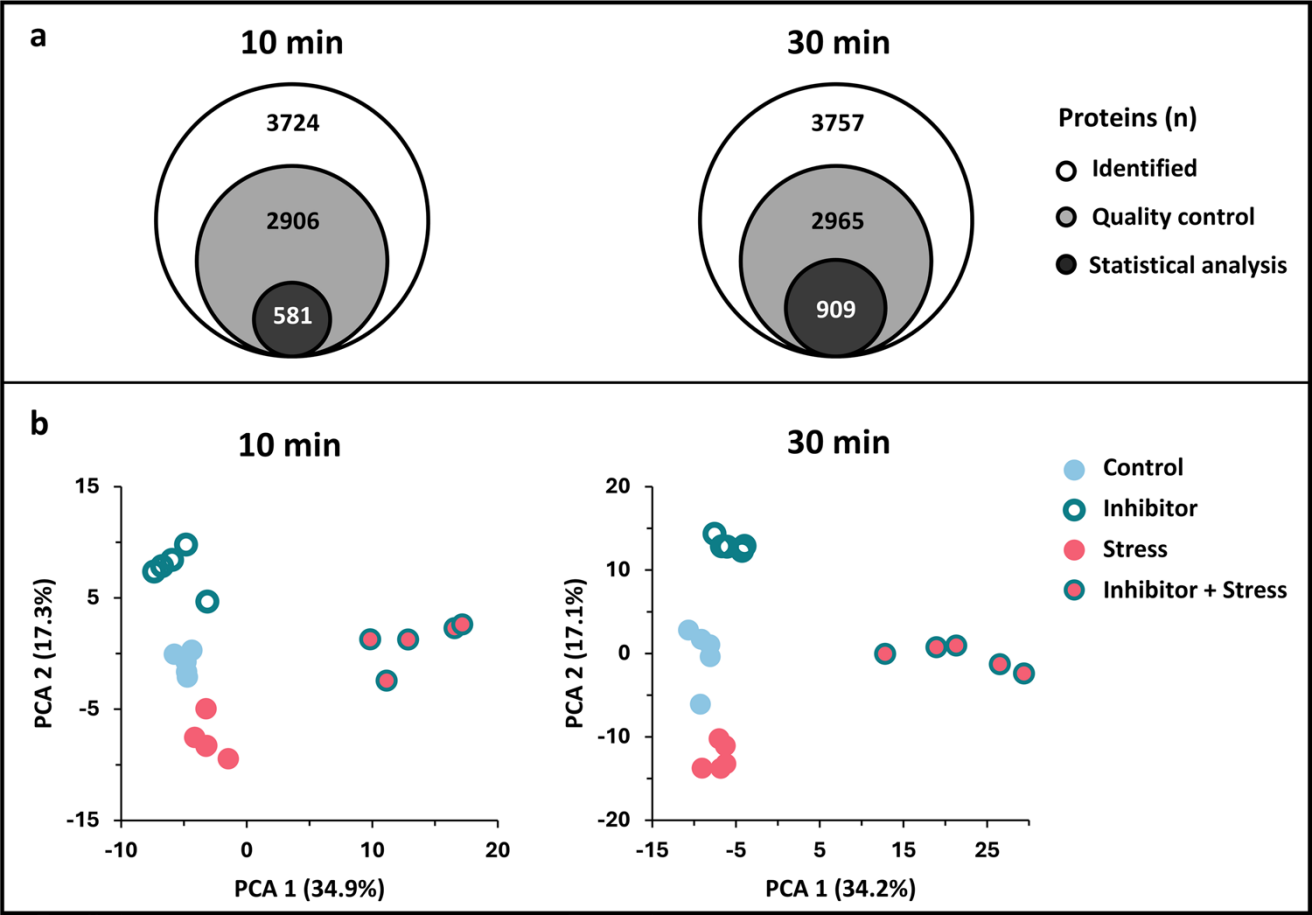
Overview of the initial data analysis for the 10 min (left) and 30 min (right) samples with **a** showing the numbers of all proteins identified (Identified), proteins left after removal of contaminants, proteins only identified by site, and reverse annotated peptides (Quality control), and proteins left after multiple sample ANOVA test (*p* value < 0.05; statistical analysis). **b** Principal component analysis (PCA) plot of the LFQ intensities of the quantified proteins with colours indicating the different treatments: blue fill = Control, magenta circle = Stress, turquoise fill = Inhibitor and magenta cycle + turquoise fill = Inhibitor + Stress

For each of the four treatments (Control, Stress, Inhibitor, and Inhibitor + Stress), five independent biological replicates, each consisting of pooled proteins from 12 rosettes, were analyzed. For further analysis, we separated the LFQ intensities in two groups: one group representing the samples harvested after 10 min, the other group representing the samples harvested after 30 min. Proteome analysis resulted in the identification of 3724 proteins for the 10 min and 3757 proteins for the 30 min samples (Fig. 2a and Supplementary Data 1).

After a quality control step, in which proteins ‘only identified by site, reverse sequences, and potential contaminants’ were filtered out, 2906 proteins remained for the 10 min samples and 2965 proteins for the 30 min samples (Fig. 2a, quality control). After multiple sample ANOVA test (*p* value < 0.05), 581 proteins remained for the 10 min samples and 909 for 30 min (Fig. 2a, statistical analysis). To ensure biological validity, stringent filtering was applied, leading to this significant dataset reduction.

Principal component analysis (PCA) of the LFQ values showed that all replicates clearly fell into their corresponding treatment group (Fig. 2b). PC1 explains more than 34% of the variance within the data for both time points, essentially separating the Inhibitor + Stress treatment from all other treatments, while PC2 (> 17% of the variance for both time points) separates the Control, Stress, and Inhibitor treatments. Overall, this analysis indicates clear proteomic changes, especially with regard to the Inhibitor + Stress samples compared to all other treatments. The other treatments cluster closer together but still remain separated from each other.

### Clustering analysis

**Fig. 3:**
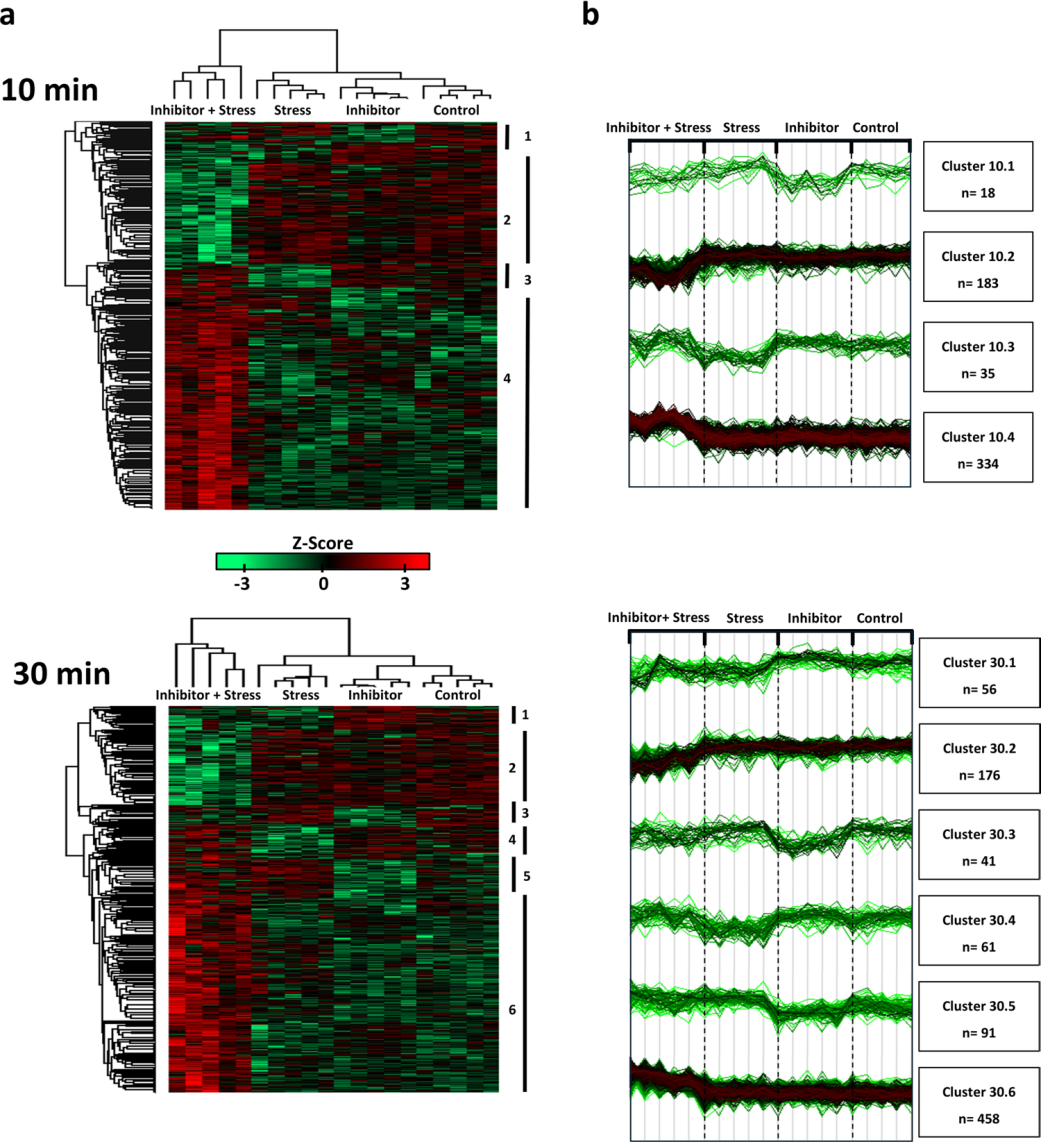
Heatmaps and hierarchical clustering of treatments and identified DAPs. **a** Heatmaps showing the z-score overall pattern of relative increased (red) and decreased (green) protein abundance within the samples after 10 (upper panel) and 30 (lower panel) min of treatment. The dendrogram of the columns (top) shows how the four different treatments separate based on Euclidean distance. The dendrogram of the rows (left side) shows the clustering of the protein. Identified protein clusters are indicated with a number on the right side of both heatmaps. **b** Intensity plots for each cluster from panel **a** and number of proteins (n) in the specific cluster is indicated

Next, hierarchical clustering of protein abundance was performed on the Z-scored normalized intensities. In line with the PCA analysis, it showed a clear clustering of all replicates of an individual treatment (Fig. 3a, dendrograms on top of the heatmaps) and therewith the strong similarity in protein abundance among the replicates within one treatment group. All five replicates of the Inhibitor + Stress treatment clustered together as one of two main clusters. In the other main cluster, two subclusters could be observed, with the first one representing the five replicates of the Stress treatment, and the second one showing a close relation of the Control and Inhibitor only treatment. This pattern of clustering was observed for both the 10 and the 30 min samples. It substantiates the strong effect of the Inhibitor + Stress treatment on the proteome, and the slightly milder but clear effect of the Stress treatment alone, while the Inhibitor treatment alone has a lesser effect. It also further substantiates an attenuating effect of Ca^2+^ signaling on the H_2_O_2_-induced stress response.

Hierarchical clustering further revealed segregation of the proteins into four different abundance clusters after 10 min of treatment, and six clusters after 30 min of treatments (Fig. 3a, dendrograms on the side of the heatmaps). The differences in protein abundance of the different samples are shown in the heatmap with green color indicating a decrease and red color an increase in relative protein abundance (Fig. 3a) as well as profile plots (Fig. 3b). The 10-min clusters were characterized by a lower protein abundance for the Inhibitor and Inhibitor + Stress treatment (cluster 10.1), a lower abundance for only the Inhibitor + Stress treatment (cluster 10.2), a lower abundance for only the Stress treatment (cluster 10.3) and a lower abundance for all but the Inhibitor + Stress treatment (cluster 10.4). The clusters identified after 30 min of treatment were defined by a lower abundance for the Inhibitor + Stress and the Stress treatment (30.1), a lower abundance for only the Inhibitor + Stress treatment (30.2), a lower abundance for only the Inhibitor treatment (30.3), a lower abundance for the only Stress treatment (30.4), a lower abundance for the Control and Inhibitor treatment (30.5), and a lower abundance for all but the Inhibitor + Stress treatment (30.6). At both time points, the clusters containing proteins with different abundance in the Inhibitor + Stress treatment only were the largest clusters, containing 183 (higher abundance; 10.2) and 334 (lower abundance; 10.4) proteins for the 10 min, and 176 (higher abundance; 30.2) and 458 proteins (lower abundance; 30.6) for the 30 min.

### Gene Ontology analysis of heatmap clusters

Proteins in each cluster were functionally classified by Gene Ontology (GO) and KEGG term enrichment analysis. Details on the different GO terms (molecular function and biological process) of all clusters are shown separately for 10 and 30 min in Tables 1 and 2, respectively.

**Table 1:** Functional classification of the protein clusters after 10 min of treatment, obtained after hierarchical clustering

Cluster	Proteins (n)	Analysis	Enrichment FDR	Protein (*n*)	Fold enrichment	Pathways
1	18	KEGG	7.40E−09	7	34.1	Ribosome
		GO Molecular function	3.60E−07	7	25.4	Structural constituent of ribosome
			1.00E−06	7	19.7	Structural molecule activity
			5.00E−05	7	10.4	MRNA binding
		GO Biological Process	5.40E−03	6	9.9	Amide biosynthetic proc
			6.30E−03	6	8.5	Cellular amide metabolic proc
			8.00E−03	7	5.9	Organonitrogen compound biosynthetic proc
2	183	KEGG	8.90E−38	40	19.2	Ribosome
			4.90E−04	5	12.4	Proteasome
			3.40E−03	4	11.4	Phenylalanine, tyrosine and tryptophan biosynthesis
		GO Molecular function	1.20E−05	3	151	MAP kinase scaffold activity
			1.20E−05	3	151	Protein kinase C binding
			1.20E−05	3	151	Signaling adaptor activity
		GO Biological Process	7.20E−35	54	9.5	Translation
			7.20E−35	54	9.4	Peptide biosynthetic proc
			1.90E−34	55	8.9	Amide biosynthetic proc
3	35	KEGG	1.0E−03	2	121.4	Sulfur relay system
			2.9E−03	2	63.1	Biosynthesis of unsaturated fatty acids
			5.7E−03	2	36.7	Alpha-Linolenic acid metabolism
		GO Molecular function	8.7E−04	2	315.7	Acetyl-CoA C-acyltransferase activity
			8.7E−04	2	315.7	Thiosulfate sulfurtransferase activity
			3.6E−03	2	105.2	Sulfurtransferase activity
		GO Biological Process	1.0E−05	4	87.7	Cysteine metabolic proc
			4.5E−06	5	52.6	Sulfur amino acid metabolic proc
			5.6E−05	7	12.6	Sulfur compound metabolic proc
4	334	KEGG	8.00E−26	23	27.6	Carbon fixation in photos. organisms
			1.50E−14	16	17.2	Glyoxylate and dicarboxylate metabolism
			4.30E−11	12	17.1	Pentose phosphate pathway
		GO Molecular function	4.10E−08	6	49.6	L-malate dehydrogenase activity
			1.40E−07	7	29	Intramolecular oxidoreductase activity, interconverting aldoses and ketoses
			1.10E−09	18	8.3	Oxidoreductase activity, acting on CH–OH group of donors
		GO Biological Process	7.60E−31	44	11.5	Response to cadmium ion
			4.70E−18	32	8.4	Nucleotide metabolic proc
			4.30E−36	78	6.1	Carboxylic acid metabolic proc

For each cluster the top three GO-terms (KEGG, molecular function, biological process), selected by FDR and sorted by fold enrichment, are displayed

**Table 2:** Functional classification of the protein clusters after 30 min of treatment, obtained after hierarchical clustering

Cluster	Proteins (*n*)	Analysis	Enrichment FDR	Protein (*n*)	Fold Enrichment	Pathways
1	56	KEGG	1.90E−03	3	24.3	Citrate cycle (TCA cycle)
			1.10E−03	4	16.6	Glycolysis / Gluconeogenesis
			1.10E−03	4	16.4	Cysteine and methionine metabolism
		GO molecular function	1.90E−04	8	9.3	Structural constituent of ribosome
			1.90E−04	9	8.1	Structural molecule activity
			4.70E−04	9	6.6	Zinc ion binding
		GO biological process	7.90E−04	4	29.5	Aspartate family amino acid metabolic proc
			2.50E−03	6	9.3	Response to cadmium ion
			2.50E−03	7	7.4	Cellular amino acid metabolic proc
2	176	KEGG	1.30E−35	38	18.9	Ribosome
			1.40E−03	4	15.3	Propanoate metabolism
			9.80E−07	9	11.8	Cysteine and methionine metabolism
		GO molecular function	1.90E−19	20	21.8	RRNA binding
			3.80E−41	46	17.1	Structural constituent of ribosome
			3.80E−41	50	14.4	Structural molecule activity
		GO biological process	6.00E−36	54	9.9	Translation
			6.10E−36	54	9.8	Peptide biosynthetic proc
			2.30E−37	57	9.6	Amide biosynthetic proc
3	41	KEGG	4.30E−02	1	224.6	Caffeine metabolism
			4.30E−02	2	17.5	Phagosome
			3.60E−02	3	12.9	Endocytosis
		GO molecular function	1.30E−03	5	16	GTPase activity
			3.70E−03	5	11.1	GTP binding
			3.70E−03	5	10.4	Guanyl nucleotide binding
		GO biological process	3.90E−02	2	84.2	Mitochondrial fission
			3.90E−02	2	74.9	Purine-containing compound catabolic proc
			3.90E−02	3	20.8	Endocytosis
4	61	KEGG	3.30E−03	3	23.8	Aminoacyl-tRNA biosynthesis
			9.30E−05	5	23.1	Biosynthesis of nucleotide sugars
			1.90E−04	5	17.3	Amino sugar and nucleotide sugar metabolism
		GO molecular function	2.90E−02	3	18.9	Aminoacyl-tRNA ligase activity
			2.90E−02	3	18.9	Ligase activity, forming carbon–oxygen bonds
			2.90E−02	3	17.9	Actin filament binding
		GO biological process	2.10E−03	3	71.5	Pentose metabolic proc
			7.60E−03	5	12	Monosaccharide metabolic proc
			2.80E−04	9	8.7	Cellular amino acid metabolic proc
5	91	KEGG	5.90E−03	6	6.8	Carbon metabolism
			2.40E−03	14	3.4	Biosynthesis of secondary metabolites
		GO molecular function	8.70E−03	3	31.4	Poly(U) RNA binding
			8.70E−03	3	27.6	Poly-pyrimidine tract binding
			8.70E−03	4	14.8	NAD binding
		GO biological process	1.00E−03	6	12.7	Photosynthesis, light reaction
			6.30E−03	7	6.7	Response to cadmium ion
			3.90E−04	11	6.5	Generation of precursor metabolites and energy
6	458	KEGG	2.70E−21	22	19.3	Carbon fixation in photosynthetic organisms
			1.90E−17	20	15.7	Glyoxylate and dicarboxylate metabolism
			5.70E−13	15	14.9	Citrate cycle (TCA cycle)
		GO molecular function	7.70E−09	8	28.4	Malate dehydrogenase activity
			4.90E−10	14	12.3	Protein domain specific binding
			5.70E−08	13	9.6	NAD binding
		GO biological process	4.40E−38	56	10.7	Response to cadmium ion
			9.90E−34	59	8.2	Response to metal ion
			1.00E−47	104	6	Carboxylic acid metabolic proc

For each cluster the top three GO-terms (KEGG, molecular function, biological process), selected by FDR and sorted by fold enrichment, are displayed

Enriched terms in three out of four clusters with a relative lower abundance of proteins in the Inhibitor + Stress treatment (10.1, 10.2 and 30.2) included ribosomal pathways. Cluster 10.2, with lower abundance only for Inhibitor + Stress, also included the KEGG term proteasome. Clusters 10.4, 30.5, and 30.6, all of which comprise a relative higher abundance of proteins in the Inhibitor-Stress treatment, have carbon fixation and other pathways related to carbon metabolisms as the most enriched KEGG terms.

### Identification of proteins with significant change in abundance (DAPs)

**Fig. 4:**
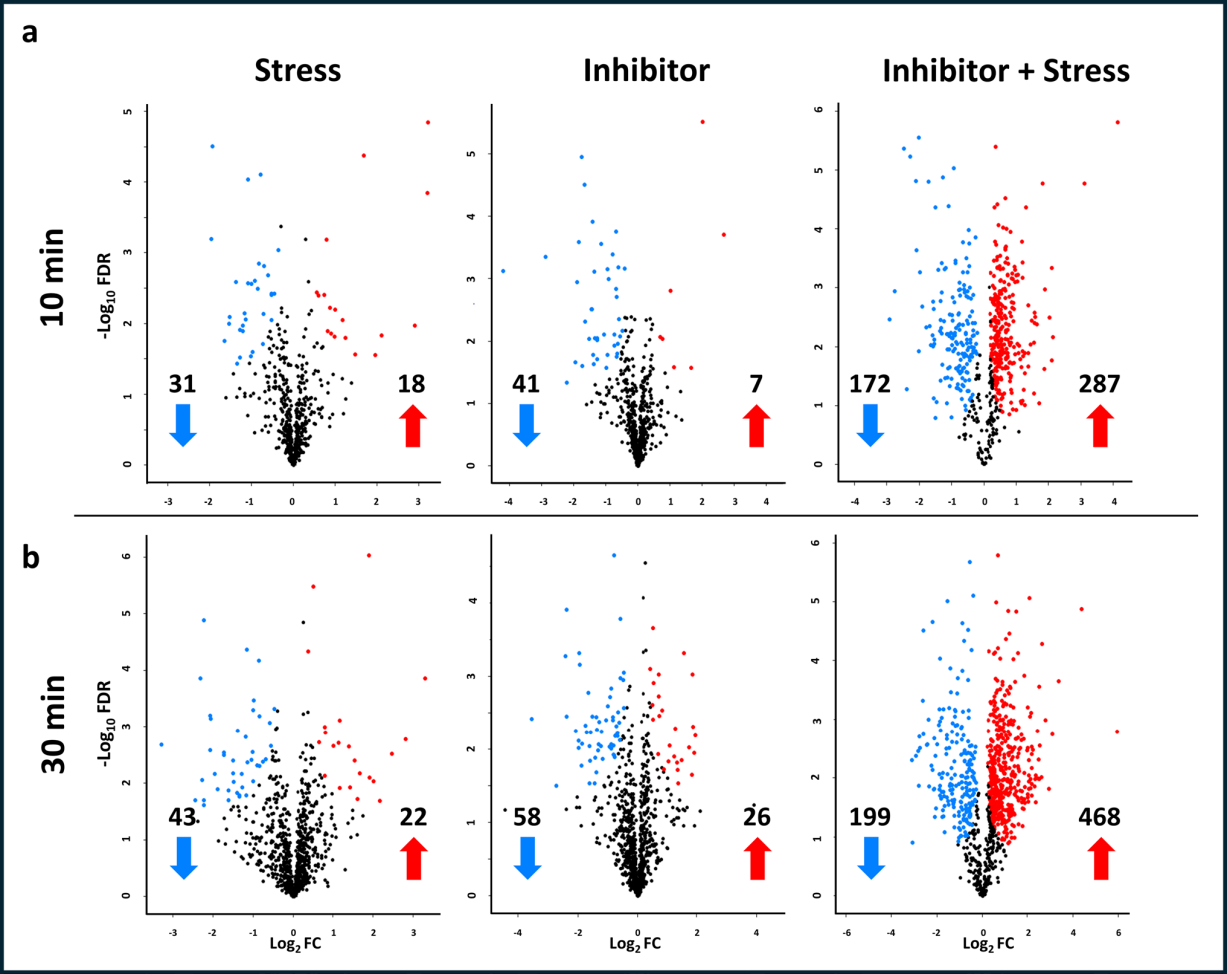
Volcano plots indicating DAPs (FDR 0.05) for three treatments (Stress, Inhibitor and Inhibitor + Stress) in comparison to control (double mock) samples after **a** 10 min and **b** 30 min of treatment**.** Proteins were graphed by fold change (Log_2_FC; *x*-axis) and the confidence statistic (−Log_10_FDR *P*; *y*-axis). Blue dots represent proteins that show a significant lower abundancy while red dots represent proteins that show a significant higher abundancy for the indicated treatment compared to control samples. Black dots represent the proteins that do not show a significant change in abundance (S0 = 0.1, FDR = 0.05)

Quantitative differences occurring among proteome profiles due to the different treatments were detected by comparing the individual protein intensities in each treatment group (Stress, Inhibitor, and Inhibitor + Stress) with the control samples. Protein abundance differences were obtained by performing a *t*-test (*p* < 0.05) on the proteins shown to be significant different in any of the treatments (Supplementary Data 2), i.e., 581 proteins after 10 min and 909 proteins after 30 min of treatment, and visualized in volcano plots (Fig. 4a and b).

This analysis resulted in 49 DAPs (18 more abundant and 31 less abundant) between Stress treatment and control after 10 min and 65 DAPs (22 more abundant, 43 less abundant) after 30 min. For the Inhibitor only treatment, a total of 48 DAPs (7 higher abundant, 41 less abundant) were found after 10 min and 84 DAPs (26 more abundant, 58 less abundant) in the 30 min set. The highest number of differences occurred for the Inhibitor + Stress treatment versus control, with 459 DAPs (287 more abundant, 172 less abundant) after 10 min and 667 DAPs (468 more abundant, 199 less abundant) after 30 min. The DAPs identified for each treatment were further subjected to comparable analysis to categorize them based on whether they require Ca^2+^ for their regulation.

### Comparison of DAPs and identification of Ca^2+^-dependent and -independent proteins

**Fig. 5:**
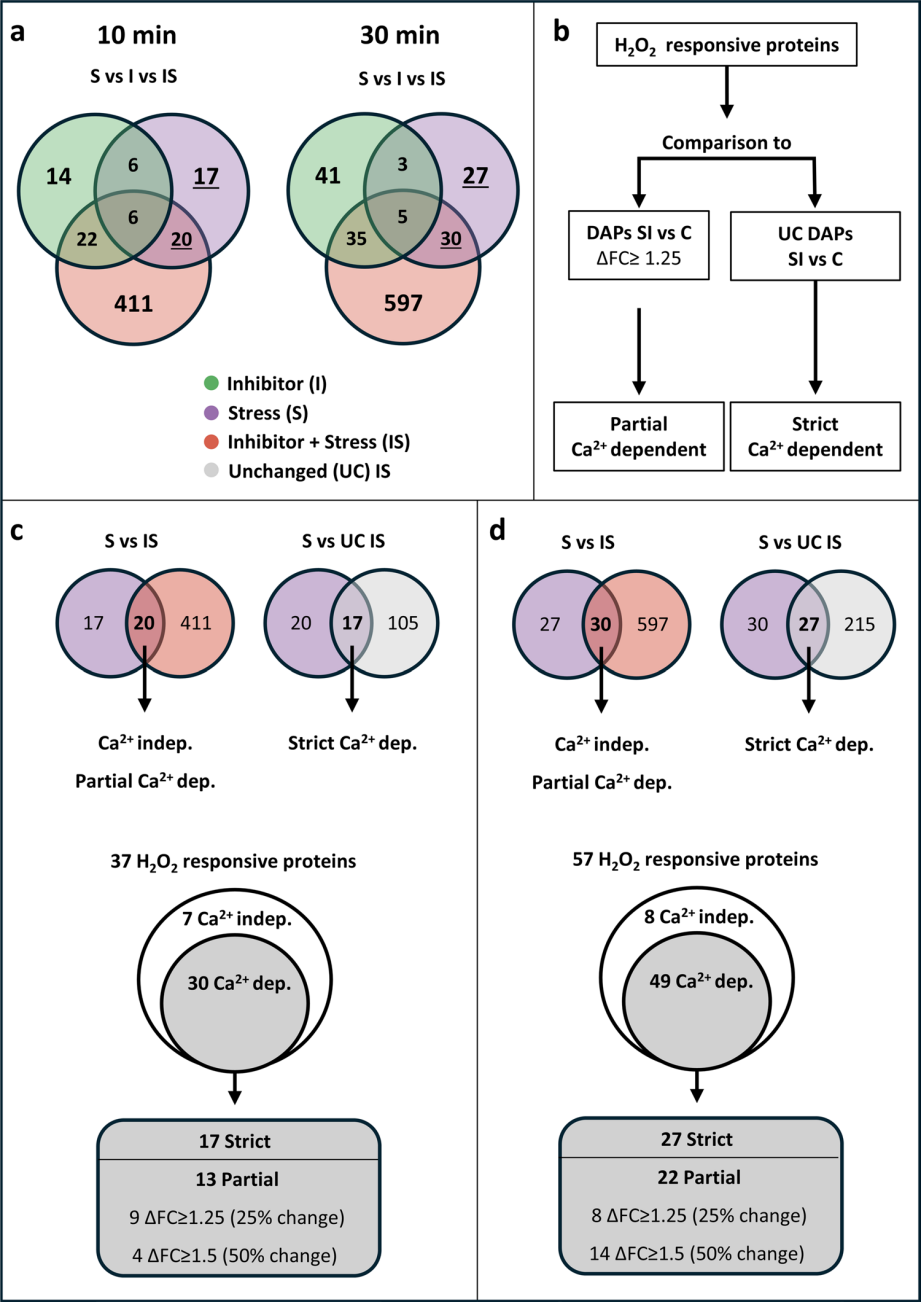
Identification and categorisation of H_2_O_2_ responsive proteins. **a** Venn diagrams showing all identified DAPs and their overlap between the different treatments. Underlined numbers are the H_2_O_2_ responsive proteins. **b** Schematic representation of the analysis steps to identify the different levels of Ca^2+^ dependency for H_2_O_2_ responsive proteins. **c** Categorisation of the H_2_O_2_ responsive proteins after 10 min of stress treatment. **d** Categorisation of the H_2_O_2_ responsive proteins after 30 min of stress treatment

After identification of the DAPs for each treatment vs control at both timepoints (Fig. 4), the DAPs of each treatment were compared to all other treatments of the same timepoint to find overlapping and unique proteins (Fig. 5a). In general, the overlapping numbers were relatively low, indicating that each treatment has an individual effect on the proteome. For both time points, the largest overlap was observed between Stress and Inhibitor + Stress as well as Inhibitor and Inhibitor + Stress with a much smaller overlap between Stress and Inhibitor. As the total number of proteins differs between the two time points, we decided to also compare the relative numbers (% from total proteins) of DAPs (supp.

l. Figure 1). This comparison shows a very similar pattern between the two time points.

For the further analysis, we decided to focus on H_2_O_2_-responsive proteins, i.e., those proteins that show a difference in abundance between H_2_O_2_ treatment and control (DAP-Stress, 10 and 30 min). From these sets, we omitted the DAPs that showed a different abundance upon treatment with LaCl_3_ alone, to avoid effects of the inhibitor that are not related to the reduction of the H_2_O_2_-induced Ca^2+^ transient. This resulted in a set of 37 and 57 H_2_O_2_-responsive proteins after 10 and 30 min of treatment, respectively (Fig. 5a, underlined numbers). These stress-responsive proteins were further categorized as being Ca^2+^-independent or Ca^2+^-dependent, depending on their abundance in the Inhibitor + Stress treatment. For this categorization, we used the method described by Bhattacharyya et al. (2025). The steps used in this approach are schematically displayed in Fig. 5b. H_2_O_2_-responsive proteins that showed an unchanged abundance (UCs) compared to control under Inhibitor + Stress treatment can be considered strictly Ca^2+^-dependent in their H_2_O_2_ response (Fig. 5c and d). Proteins that showed a differential abundance upon both Stress vs. control and Stress + Inhibitor vs. control, but their abundance level differed significantly (ΔFC ≥ 1.25, corresponding to a change in protein abundance of at least 25%) between the two treatments, were categorized as being partially Ca^2+^-dependent (Fig. 5c and d). This group was further split in two categories, with the lower threshold set to a change in abundance of at least 25% up to 50%, and the higher threshold including all proteins showing a change in abundance greater than 50% (ΔFC ≥ 1.5). Proteins that showed no differential abundance between Stress and Stress + Inhibitor treatment (ΔFC ≤ 1.25) were considered Ca^2+^-independent in their H_2_O_2_ response.

This analysis identified a total of 7 Ca^2+^-independent and 30 Ca^2+^-dependent H_2_O_2_-responsive proteins after 10 min of treatment (Fig. 5c). Among the Ca^2+^-dependent proteins, 17 were classified as strictly Ca^2+^-dependent and 13 partial Ca^2+^-dependent. Of these 13 partial Ca^2+^-dependent proteins, 4 met the threshold of at least a 50% change in abundance (≥ 1.5-fold increase or 0.5-fold ≥ decrease).

For the 30 min treatment, 8 Ca^2+^-independent and 49 Ca^2+^-dependent H_2_O_2_-responsive proteins were identified (Fig. 5d). Within the Ca^2+^-dependent group, 27 proteins were strictly Ca^2+^-dependent, a similar percentage as observed at 10 min, while 22 proteins displayed partial Ca^2+^-dependence. Among the partially Ca^2+^-dependent proteins, 14 met the threshold of at least a 50% change in abundance (≥ 1.5-fold increase or 0.5 ≥ decrease). A detailed list of the H_2_O_2_-responsive proteins, along with their classification as Ca^2+^-independent, partially Ca^2+^-dependent or strictly Ca^2+^-dependent, is provided in Table 3 for the 10 min and Table 4 for the 30 min data set.

**Table 3:** List of H_2_O_2_-responsive proteins indicated as strict Ca^2+^-dependent, partial Ca^2+^-dependent, and Ca^2+^-independent after 10 min of treatment

		Protein ID	Abundancy compared to control	Full name	ΔFC (SvsC-SIvsC)	Symbol
Strict Ca^2+^-dependent		AT1G16460	Less	Sulfurtransferase 2		STR2
		AT1G48350	More	Large ribosomal subunit protein uL18c		RPL18
		AT1G62180	Less	5′ Adenylylsulfate reductase 2, chlorplastic		APR2
		AT1G72610	More	Germin-like protein		GLP1
		AT2G30520	More	Root phototropism protein 2		RPT2
		AT2G44060	Less	Late embryogenesis abundant protein		LEA26
		AT3G07660	Less	Flocculation protein		DUF1296
		AT3G14990	Less	Protein DJ-1 homolog A		DJ1A
		AT3G17880	Less	TPR repeat-containing thioredoxin		TDX
		AT3G17900	Less	Heat-inducible transcription repressor		MEB5_12
		AT3G45850	Less	Kinesin motor domain-containing protein		KIN5D
		AT3G53260	Less	Phenylalanine ammonia-lyase 2		PAL2
		AT4G28730	Less	Glutaredoxin-C5 chloroplastic		GRXC5
		AT5G06460	Less	Ubiquitin-activating enzyme		UBA2
		AT5G21274	Less	Calmodulin		CAM
		AT5G43830	Less	Domain containing protein		DUF3700
		AT5G59890	Less	Actin-depolymerizing factor 4		ADF4
Partial Ca^2+^-dependent	ΔFC ≥ 1.5	AT1G13750	More	Probable inactive purple acid phosphatase 1	5.37	PAP1
		AT1G20160	More	CO(2)-response secreted protease	2.51	CRSP
		AT3G49080	Less	Small ribosomal subunit protein uS9m	1.71	RPS9M
		AT4G38510	More	V-type proton ATPase sub-unit B2	2.34	VHA-B2
	ΔFC 1.25–1.5	AT1G10200	Less	LIM domain-containing protein	1.26	WLIM1
		AT1G14030	More	Fructose-bisphosphate aldolase	1.39	LSMT-L
		AT1G26460	More	Tetratricopeptide repeat-containing protein	1.32	TPR
		AT1G70890	Less	MLP-like protein 43	1.45	MLP43
		AT2G20890	More	Thylakoid formation 1	1.29	THF1
		AT3G53130	More	Carotene epsilon-monooxygenase, chloroplastic	1.29	CYP97C1
		AT4G11260	Less	SGT1 homolog B	1.40	SGT1B
		AT4G14440	More	Enoyl-CoA delta isomerase 3	1.28	ECI3
		AT4G29510	Less	Protein arginine *N*-methyltransferase 1.1	1.34	PRMT11
Ca^2+^-independent	ΔFC < 1.25	AT1G11790	Less	Arogenate dehydratase	1.16	ADT1
		AT2G20900	More	Diacylglycerol kinase 5	1.11	DGK5
		AT2G42690	More	Phospholipase A1-IIdelta	1.07	AGAP1
		AT3G45140	More	Lipoxygenase 2, chloroplastic	1.10	LOX2
		AT4G13010	More	Chloroplast envelope quinone oxidoreductase homolog	1.13	CEQORH
		AT4G28660	More	Photosystem II reaction center Psb28 protein	1.00	PSB28
		AT5G63870	Less	Serine/threonine protein phosphatase 7	1.09	PP7

Partial Ca^2+^-dependent proteins were further divided by a threshold of 50% change in abundance (≥ 1.5-fold increase or ≥ 0.5-fold decrease)

**Table 4:** List of H_2_O_2_-responsive proteins indicated as strict Ca^2+^-dependent (dark grey), partial Ca^2+^-dependent (light grey) and Ca^2+^-independent (white) regulated after 30 min of treatment

		Protein ID	Abundancy compared to control	Full name	Δlog_2_FC (SvsC-SIvsC)	Symbol
Strict Ca^2+^-dependent		AT1G43140	Less	Putative cullin-like protein		CUL
		AT1G10200	Less	LIM domain-containing protein		WLIM1
		AT1G16340	Less	2-Dehydro-3-deoxyphosphooctonate		KDSA2
		AT1G16460	Less	Sulfurtransferase 2		STR2
		AT1G21065	Less	Secondary thiamine-phosphate synthase enzyme		T22I11.11
		AT1G26880	Less	Large ribosomal subunit protein		RPL34A
		AT1G35580	Less	Alkaline/neutral invertase		CINV1
		AT1G55450	More	Methyltransferase		
		AT1G69250	Less	Nuclear transport factor 2		NTF2
		AT1G75660	Less	5′–3′ Exoribonuclease 3		XRN3
		AT2G02100	More	Defensin-like protein 2		PDF2.2
		AT2G29700	Less	Pleckstrin homology domain-containing protein 1		PH1
		AT3G15090	Less	GroES-like zinc-binding alcohol dehydrogenase fam		
		AT3G16050	Less	Pyridoxal 5′-phosphate synthase-like subunit PDX1.2		PDX12
		AT3G43540	Less	Initiation factor 4F subunit		DUF1350
		AT3G54170	Less	FKBP12-interacting protein of 37 kDa		FIP37
		AT3G57870	Less	SUMO-conjugating enzyme		SCE1
		AT4G01883	Less	Polyketide cyclase/dehydrase and lipid transport protein		MLBP1
		AT4G23650	More	Calcium-dependent protein kinase 3		CPK3
		AT4G36020	Less	Cold shock protein 1		CSP1
		AT5G01600	Less	Ferritin-1, chloroplastic		FER1
		AT5G11810	Less	Rhomboid family protein		T22P22_200
		AT5G18100	Less	Superoxide dismutase		CSD3
		AT5G53530	Less	Vacuolar protein sorting-associated protein 26A		VPS26A
		AT5G57890	Less	Anthranilate synthase beta subunit 2, chloroplastic		ASB2
		AT5G62340	More	Pectin methylesterase inhibitor superfamily protein		MMI9.17
		ATCG00270	More	Photosystem II D2 protein		PSBD
Partial Ca^2+^-dependent	ΔFC ≥ 1.5	AT1G03030	More	RING1B	4.58	
		AT1G56700	More	Pyrrolidone-carboxylate peptidase	4.81	
		AT1G60950	More	Ferredoxin	1.82	FD2
		AT1G74060	Less	Large ribosomal subunit protein L6y-2	1.71	RPL6B
		AT2G17870	Less	Cold shock domain-containing protein 3	1.66	CSP3
		AT2G18020	Less	Large ribosomal subunit protein uL2z	1.69	RPL8A
		AT2G32500	More	Sucrose-phosphatase	1.66	
		AT2G42690	More	Phospholipase A1-IIdelta	2.13	AGAP1
		AT3G02830	Less	Zinc finger CCCH domain-containing protein 33	2.46	ZFN1
		AT3G07630	Less	Arogenate prephenate dehydratase 2, chloroplastic	1.58	ADT2
		AT3G54440	More	Glycoside hydrolase family 2 protein	3.71	
		AT4G12730	Less	Fasciclin-like arabinogalactan protein 2	2.04	FLA2
		AT4G33220	Less	Probable pectinesterase/pectinesterase inhibitor 44	2.03	PME44
		AT5G54430	More	Adenine nucleotide alpha hydrolases-like superfamily protein	2.14	PHOS32
	ΔFC 1.25–1.5	AT1G20110	Less	Protein FREE1	1.49	FREE1
		AT1G52380	Less	Nuclear pore complex protein NUP50A	1.27	NUP50A
		AT1G70890	Less	MLP-like protein 43	1.35	MLP43
		AT1G79750	More	NADP-dependent malic enzyme	1.40	NADP-ME4
		AT2G33040	More	ATP synthase subunit gamma, mitochondrial	1.28	ATPC
		AT3G50440	More	Methylesterase 10	1.47	MES10
		AT5G38520	More	Alpha/beta-Hydrolases superfamily protein	1.39	CLD1
		AT5G66030	More	Golgi-localized GRIP domain-containing protein	1.47	GRIP
Ca^2+^-independent	ΔFC < 1.25	AT1G71220	More	UDP-glucose:glycoprotein glucosyltransferases	1.14	UGGT
		AT3G45140	More	Lipoxygenase 2, chloroplastic	1.14	LOX2
		AT3G53130	More	Carotene epsilon-monooxygenase, chloroplastic	1.06	CYP97C1
		AT4G01690	More	Protoporphyrinogen oxidase	1.17	PPOX1
		AT4G02230	Less	Large ribosomal sub-unit protein eL19y	1.22	RPL19C
		AT4G04210	Less	Plant UBX domain-containing protein 4	1.11	PUX4
		AT5G38430	More	Ribulose bisphosphate carboxylase small chain 1B, chloroplastic	1.13	RBCS-1B
		AT5G58060	More	VAMP-like protein YKT61	1.21	YKT61

Partial Ca^2+^-dependent proteins were further divided by a threshold of at least a 50% change in abundance (≥ 1.5-fold increase or 0.5-fold ≥ decrease)

Among the proteins listed in Tables 3 and 4, several belong to functional categories previously associated with Ca^2+^- or ROS-related stress, such as the ribosomal protein RPL18, the germin-like protein GLP1, calmodulin and the Ca^2+^-dependent protein kinase CPK3, which have established roles in stress signaling, redox regulation, or Ca^2+^-mediated pathways. These proteins represent key-candidates for Ca^2+^-dependent modulation of early oxidative stress responses but also illustrate the diversity of early H_2_O_2_-responsive targets.

### Functional analysis of Ca^2+^-dependent and -independent H_2_O_2_-responsive proteins

**Fig. 6:**
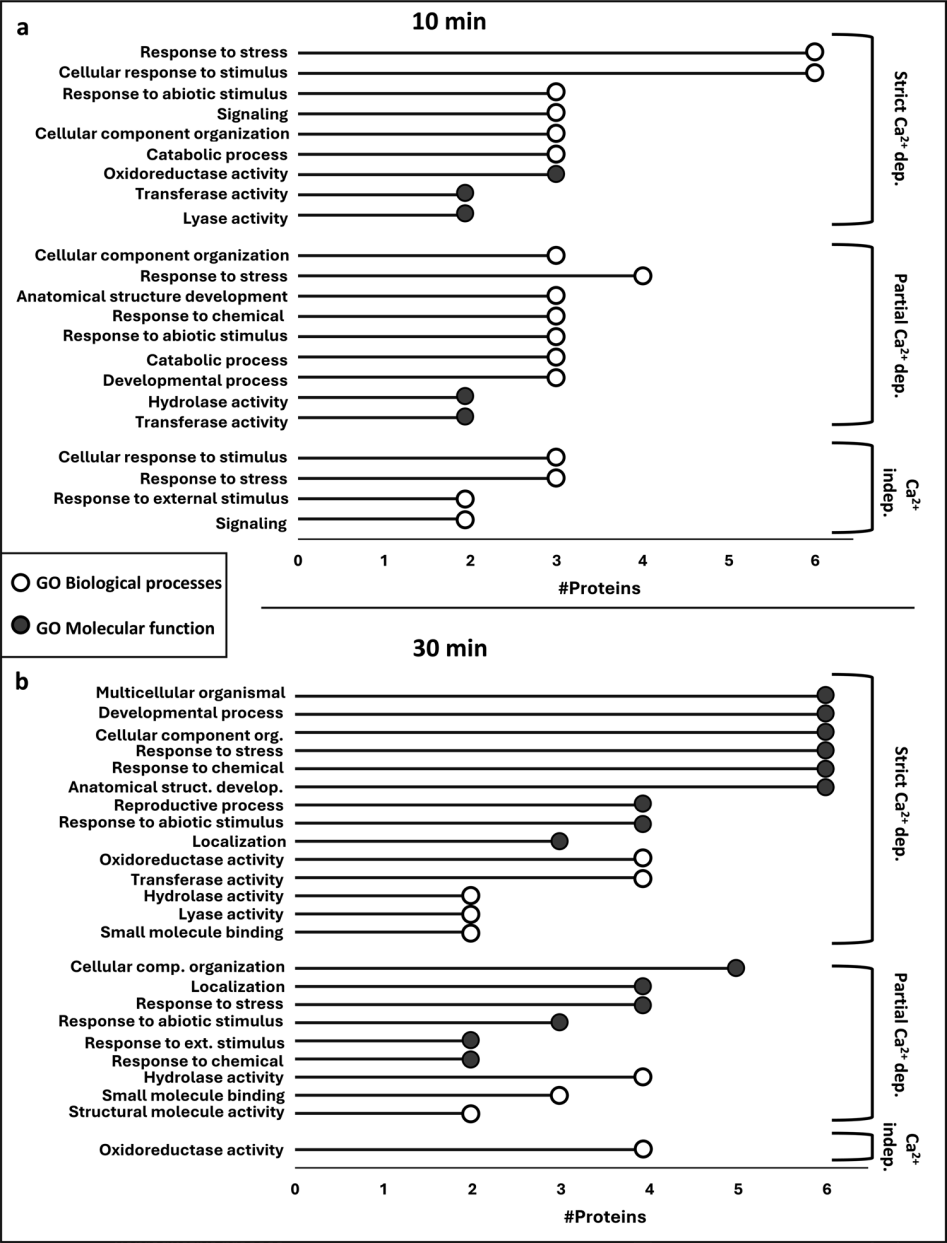
GO term analysis (biological processes and molecular function) on the H_2_O_2_-responsive proteins indicated as strict Ca^2+^-dependently, partial Ca^2+^-dependently, and Ca^2+^-independently regulated after (**a**) 10 and (**b**) 30 min of treatment. Numbers represent absolute numbers of proteins that are annotated to functional categories based on high-level GO terms

GO enrichment analysis was performed on the proteins identified as Ca^2+^-dependently and -independently H_2_O_2_-responsive (Fig. 6). As we have a rather low number of DAPs, classical enrichment analysis might lead to over-interpretation of the data, therefore, we focused on grouping the proteins by functional categories that are defined by high-level GO terms from the database containing the GO terms for biological processes (white) or molecular function (gray). More detailed information about the proteins leading to the identified GO functional category can be found in Supplementary Tables S1 and S2.

Irrespective of their Ca^2+^ dependency, H_2_O_2_-responsive proteins found after 10 min were most often associated with biological processes related to stress (Fig. 6a). After 30 min, however, biological processes and molecular functions related to development, anatomy, or cellular composition became more abundant (Fig. 6b). This suggests that after an immediate stress reception response, appropriate cellular changes are implemented. However, overall, no clear distinction between the Ca^2+^-dependent and -independent H_2_O_2_-responsive proteins can be made regarding the biological processes they are involved in. Therefore, further investigations at the individual protein level, including their specific roles in stress responses, are necessary to gain deeper insights into Ca^2+^-dependent and -independent H_2_O_2_-related regulatory mechanisms.

### Effect of the duration of the stress treatment

**Fig. 7:**
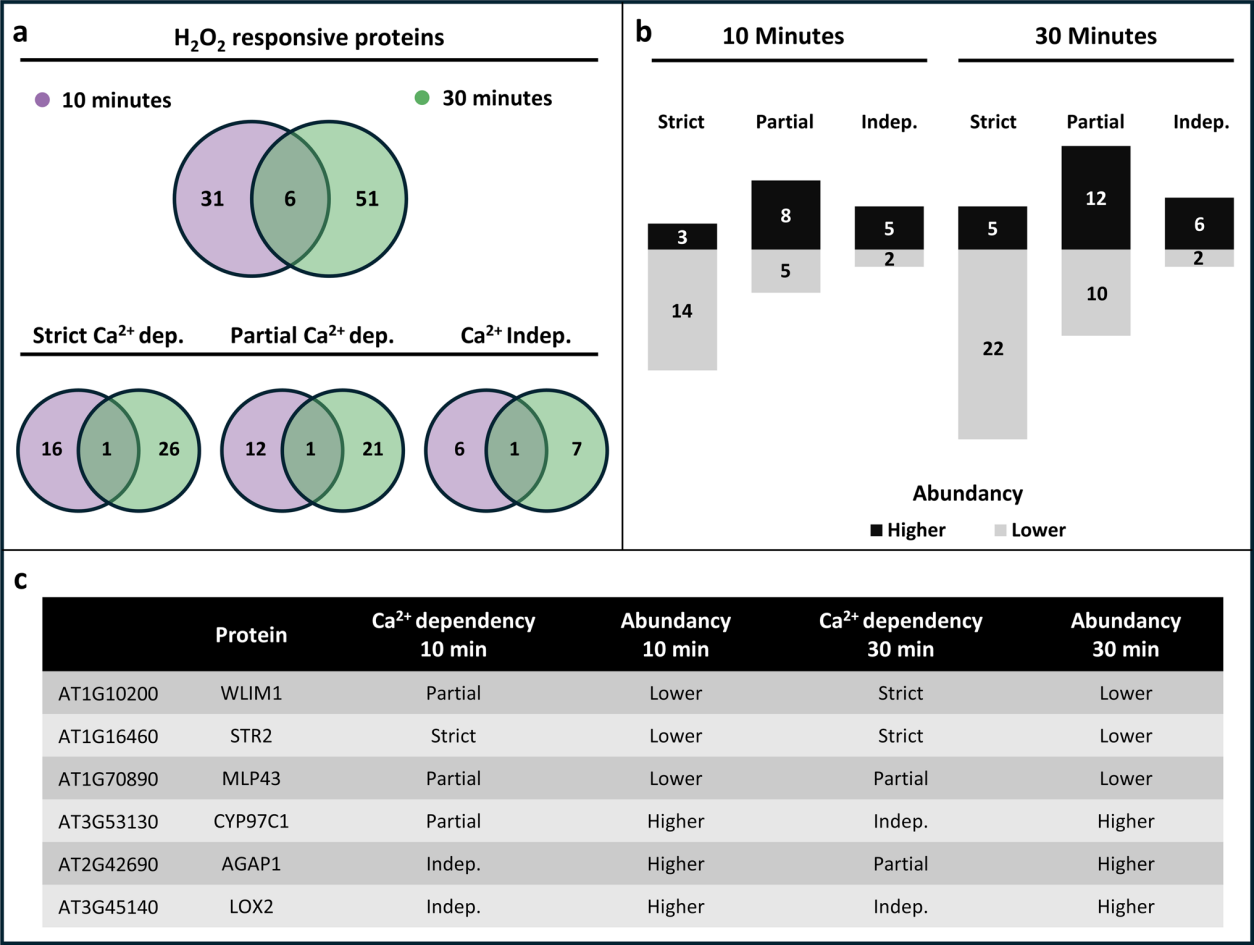
Comparison of the number of H_2_O_2_ responsive proteins between 10 and 30 min of stress treatment. **a** Venn diagrams for comparison of H_2_O_2_ responsive proteins identified after 10 min (purple) and 30 min (green) of stress treatment considering all proteins (top part) as well as separated by their dependency on Ca^2+^ (bottom). **b** Bar chart showing numbers of higher and lower abundant DAPs in the different groups of Ca^2+^ dependency. **c** Ca^2+^ dependency and the change in abundancy of six H_2_O_2_ responsive proteins found in 10 and 30 min of stress treatment

As stated above, the largest difference in biological function was observed between the two time points of stress treatment. To further elucidate the effects of stress duration on DAPs, a comparative analysis was conducted using two approaches. First, the absolute numbers of H_2_O_2_-responsive proteins identified after 10 and 30 min of stress treatment were compared. This comparison, visualized in a Venn diagram (Fig. 7a, upper panel), revealed a duration-related increase of H_2_O_2_-responsive proteins from 37 to 57, with an overlap of only six proteins between the two time points. The latter is in line with the different biological processes observed for the proteins in the two data sets (Fig. 6a and b) and indicates that the duration of the treatment results in a significant different effect on the proteome. The same trend holds true, when proteins were separated based on their Ca^2+^-dependency (Fig. 7a, lower panel).

We further investigated the effect of stress duration by additionally considering the increase and decrease of proteins abundance (Fig. 7b). Although the total number of DAPs was relatively low, a clear pattern emerged. Proteins that were strictly Ca^2+^-dependent predominantly exhibited a significant decrease in abundance, whereas those that were partially Ca^2+^-dependent or Ca^2+^-independent tended to show increased abundance. Initial LFQ values in the control samples between 10 and 30 min were nearly identical for these proteins (Supplementary Data 1), confirming that the difference is caused by the stress treatment.

We then had a closer look at the only six H_2_O_2_-responsive proteins that were identified in both time points (Fig. 6a, upper panel). Their Ca^2+^-dependency and changes in abundance were examined across both conditions (Fig. 6c). The analysis revealed that all six proteins exhibited the same change in abundance (either lower or higher) after both 10 and 30 min of stress treatment. The proteins covered all types of Ca^2+^-dependency, with three proteins falling consistently into the same group. However, the other three proteins, WLIM1, CYP97C1, and AGAP1, displayed a difference in their Ca^2+^-dependency between the two time points going partial to strict, partial to independent, and independent to partial, respectively.

### Validation of the dataset

**Fig. 8:**
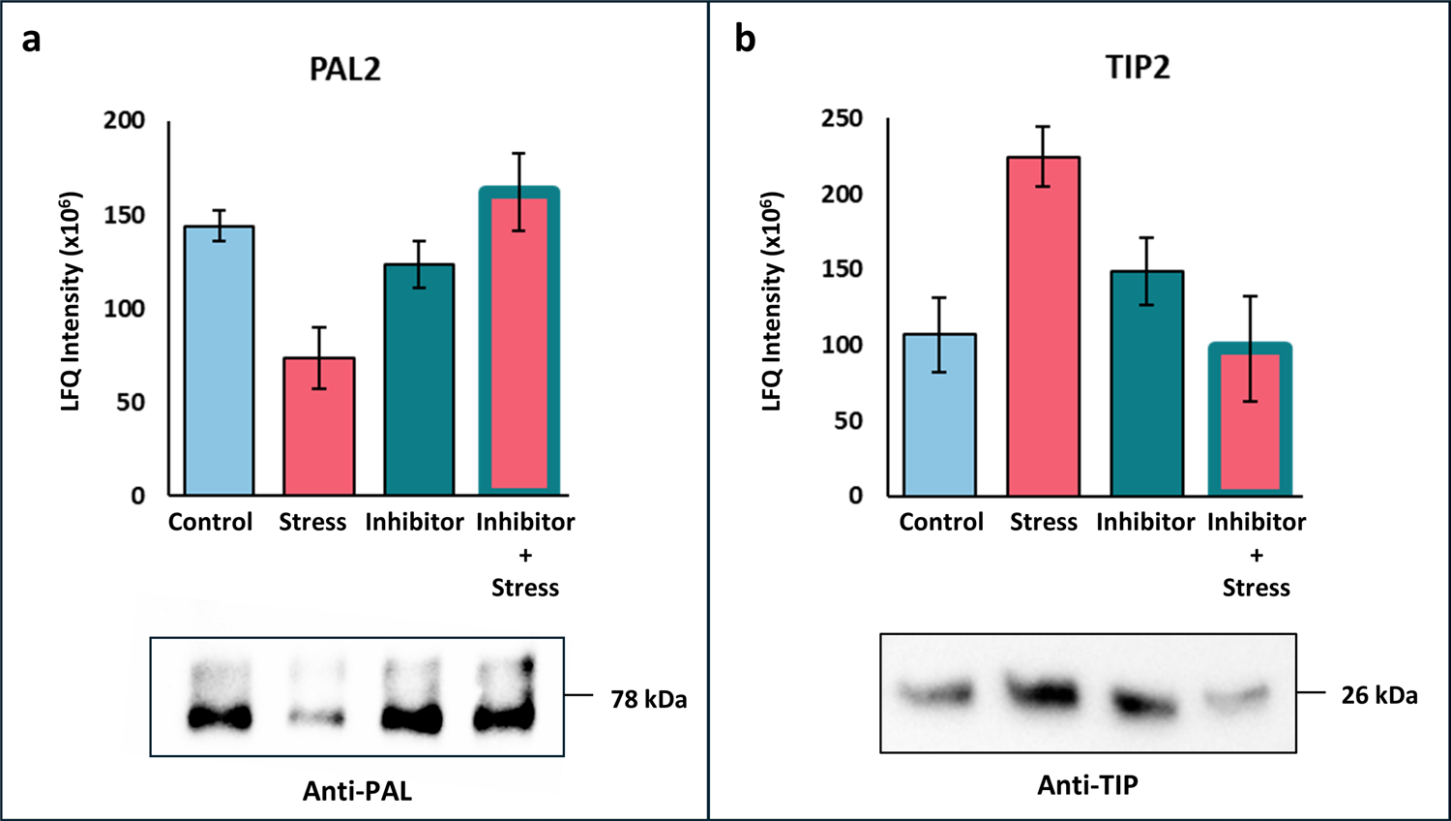
Western blot validation of proteomic data. Averages (*n* = 5) of the raw LFQ intensity values (bar graph) and immunodetection using specific antibodies for (blot) for **a** PAL2 and **b** TIP2. Full sized blots are shown in Supplementary Fig. S2

We tried to assess the accuracy of the mass spectrometric analysis by comparing the data output (raw average LFQ intensity values) with western blot analysis for two proteins from our dataset, for which antibodies could be obtained (Fig. 8). PHENYLALANINE LYASE 2 (PAL2), known to be responsive to oxidative stress (Stanley Kim et al. 2005), showed a lower abundance after 10 min of H_2_O_2_ treatment (Fig. 8a, upper panel) and was found in the group of strictly Ca^2+^-dependent H_2_O_2_-responsive proteins (Table 3). The aquaporin GAMMA TONOPLAST INTRINSIC PROTEIN 2 (TIP2), suspected to be involved in hydrogen peroxide transmembrane transport (Bienert et al. 2007), showed a higher abundance in the 10 min stress treated samples compared to control but is not in Table 4 due to its also higher LFQ values after inhibitor only treatment (Fig. 8b upper panel). In both cases, the pattern of abundance change could be confirmed by the western blot analysis (Fig. 8a and b, lower panel), where the protein band detected in the extracts of stress-treated plant material was either fainter (PAL2) or stronger (TIP2) as for the other treatments. While only shown for two candidates, this agreement between the proteome data (LFQ values) and the western blot analysis strongly validates the proteomic results.

## Discussion

In this study, we employed a pre-treatment of *Arabidopsis* rosette leaves with the Ca^2+^ channel inhibitor LaCl_3_ to differentiate between Ca^2+^-dependent and Ca^2+^-independent, short-term changes in protein abundance after treatment with H_2_O_2_ at a proteome-wide scale. We detected a total of 3724 and 3757 protein after 10 min and 30 min of treatment, respectively, well in line with the number of proteins identified in previous proteomics analysis in *Arabidopsis* (Seaton et al. 2018; Ayash et al. 2021; Scholz et al. 2025). While we refer to proteins, our analysis is based on protein groups, as reported by the MaxQuant/Perseus workflow. This grouping reflects the fact that many peptides are shared between highly similar isoforms or homologous proteins and thus avoids over-interpretation of ambiguous peptide assignments. While this means that not all identifications can be unambiguously linked to a single gene product, reporting protein groups increases the reliability of the dataset by ensuring that only well-supported identifications are considered (Cox and Mann 2008; Tyanova et al. 2016a, b; The et al. 2022). With regard to peptide numbers and coverage values, comparative values have been reported in previous large-scale proteomics studies in Arabidopsis, supporting the robustness of our dataset (Seaton et al. 2018; Ayash et al. 2021; van Wijk et al. 2021; Scholz et al. 2025). Proteomics experiments often generate a large number of low-confidence identifications or proteins with inconsistent quantifications across replicates (Al Shweiki et al. 2017; Wryk et al. 2024), so after rigorous quality control, 581 and 909 proteins were defined in the 10 and 30 min samples, respectively, that significantly changed abundance. The higher number of differentially abundant proteins detected at 30 min compared to 10 min suggests that prolonged oxidative stress triggers a wider range of adaptive processes, including enhanced synthesis (increased abundance) or protein turn-over and targeted degradation of (stress-related) proteins (decreased abundance).

A key initial step in our analysis was the identification of H_2_O_2_-responsive proteins, which resulted in distinct subsets of proteins detected after 10 and 30 min of stress treatment, with only six proteins identified in both sets. This temporal variation in protein abundance highlights the dynamic nature of the oxidative stress response and suggests that the duration of stress exposure significantly influences proteome-wide adaptations. Given that H_2_O_2_ is known to induce Ca^2+^signals at the cellular level (Rentel and Knight 2004), which are then decoded by Ca^2+^-binding proteins to activate downstream molecular processes (Mohanta et al. 2019; Tang et al. 2020), the observed temporal differences are in line with the described spatio-temporal plasticity of Ca^2+^signaling (Boulware and Marchant 2008). Consequently, it is not unexpected that a threefold increase in stress duration results in distinct proteomic responses, reflecting the dynamic and evolving nature of oxidative stress adaptation at the molecular level.

Following the identification of H_2_O_2_-responsive proteins, we further categorized them based on their dependence on Ca^2+^ for differential abundance regulation. Strict Ca^2+^ dependency was defined by proteins that exhibited significant changes in abundance upon H_2_O_2_ treatment but lack this response when pre-incubated with LaCl_3_, indicating a complete reliance on Ca^2+^ signaling for their regulation. This was the largest group for both the 10 and 30 min time point. Partially Ca^2+^-dependent proteins showed a difference in abundance both between control and H_2_O_2_ treatment as well as control and H_2_O_2_ + LaCl_3_ treatment_,_ however, the abundance was significantly different between the two treatments. Ca^2+^ independency, no significant difference in abundance between the H_2_O_2_ and the H_2_O_2_ + LaCl_3_ treatment was observed for less than 20% at both timepoints, showing the strong impact of Ca^2+^ signaling on the oxidative stress response that was also observed in a recent transcriptomic analysis on barley (Bhattacharyya et al. 2025). Another notable finding related to this was the high number of DAPs identified when the H_2_O_2_-induced Ca^2+^ transient was blocked by LaCl_3_. With over 400 DAPs at 10 and over 600 DAPs at 30 min, the numbers were about a factor 10 higher than for the stress treatment alone. This high number of DAPs in response to the combined treatment suggest that Ca^2+^ signaling can strongly attenuate the H_2_O_2_ response.

Another interesting result is the observation that much more proteins with a strict Ca^2+^ dependency show a reduced abundance, while a higher abundance is more often observed for Ca^2+^ independent H_2_O_2_-responsive proteins. Considering the timeframe of the experiment, it seems likely that protein loss is mostly driven by degradation (and not reduced transcription) while increase in protein content is the result of increased transcription and/or translation. These findings indicate a potential regulatory mechanism in which protein destabilization is driven by Ca^2+^ signaling, while proteins without strict Ca^2+^ dependency may undergo enhanced synthesis under oxidative stress conditions.

Comparison of H_2_O_2_-responsive proteins between 10 and 30 min of treatment revealed only six overlapping proteins, all of which exhibited the same change in abundance at both time points. Notably, three proteins exhibited a shift in Ca^2+^ dependency between the two time points. However, they went from partial to strictly Ca^2+^ dependent or from partial to Ca^2+^ independent, but no protein changed from strictly Ca^2+^-dependent to Ca^2+^-independent or vice versa. Since the difference in logFC change between the two time points is quite consistent for all three proteins over the five biological replicates analyzed, these findings clearly suggest a dynamic nature of Ca^2+^ dependent and independent protein regulation in response to oxidative stress.

Overall, it remains challenging to draw a definitive conclusion regarding the precise role of Ca^2+^ signaling in shaping proteomic changes from our data. However, we found several candidates with known functions in stress responses among the H_2_O_2_-responsive proteins. These H_2_O_2_-responsive proteins are involved in a variety of biological processes, such as catabolic and metabolic processes, or various transferase, hydrolase, lyase and oxidoreductase activity, pointing out the diverse cellular pathways potentially affected by Ca^2+^-dependent and independent signaling.

In all of these cases, single proteins and not protein groups were identified. The ribosomal protein RPL18, which exhibited higher abundance after 10 min of H_2_O_2_ treatment, has been previously described as a positive regulator of powdery mildew resistance in wheat (Tao et al. 2024). This observation aligns with the well-established role of ROS in plant immunity, as ROS are rapidly produced upon pathogen attack and contribute to defense responses, including cell wall reinforcement, localized cell death, and activation of downstream signaling pathways (Li et al. 2023; Wang et al. 2024). The H_2_O_2_-induced accumulation of RPL18 may therefore reflect a convergence between oxidative stress signaling and biotic defense mechanisms, suggesting that early ROS bursts could support protein synthesis pathways involved in pathogen resistance. The germin-like protein GLP1, which also showed increased abundance after 10 min, has been characterized as an oxidative stress defense enzyme in plants (Shahwar et al. 2023) and also the DJ-1 homolog A (DJ1A), showing a lower abundance after 10 min, has been described as a key protein involved in the oxidative stress response of Arabidopsis (Xu et al. 2010). CPK3, a calcium-dependent protein kinase, displayed higher abundance with strict Ca^2+^ dependency after 30 min, aligning with its known role in Ca^2+^-dependent signaling pathways involved in responses to abiotic and biotic stresses (Mehlmer et al. 2010) and plant immunity (Lu et al. 2020). For the identified H_2_O_2_-responsive proteins that lack a well-defined role in plant stress responses, further investigations are required to elucidate their functions in plant defense mechanisms, signaling pathways, ROS homeostasis, and overall plant survival.

## Conclusion

Hydrogen peroxide (H_2_O_2_) is a crucial ROS, generated as a toxic by-product of biological metabolic processes while also functioning as a signaling molecule that regulates plant growth and development. Additionally, it interacts with signaling pathways involving second messengers such as Ca^2+^. Our findings expand the current knowledge of oxidative stress responses by identifying proteins which are degraded or synthesized in response to H_2_O_2_ in a Ca^2+^ dependent manner. In these subsets, proteins were found that are known to play a role in Ca^2+^ signaling and stress response, but also proteins that are so far unassociated with stress response pathways. These novel proteins present potential targets for further investigation into the molecular basis of H_2_O_2_-Ca^2+^ interactions. Given that both biotic and abiotic stress factors can induce H_2_O_2_ accumulation and Ca^2+^ fluctuations, understanding this cross-talk is essential for deciphering plant stress acclimation mechanisms. Due to the conservation of many signaling pathways between plant species (Waszczak et al., 2018; Fichman et al. 2023), the insight gained here from Arabidopsis may have broader applications, potentially for strategies to enhance stress resilience in economically significant crop species.

## Supplementary Information

Below is the link to the electronic supplementary material.

Supplementary file1 (PDF 234 KB)

Supplementary file2 (XLSX 3800 KB)

Supplementary file3 (XLSX 88 KB)

## Data Availability

The authors upon reasonable request can provide the data presented in this study. The MS data have been deposited to the ProteomeXchange Consortium via the PRIDE (Perez-Riverol et al. 2025) partner repository with the dataset identifier PXD069039.
