# Correction: With or without a Ca^2+^ signal?: a proteomics approach toward Ca^2+^‑dependent and ‑independent changes in response to oxidative stress in *Arabidopsis thaliana*

**DOI:** 10.1007/s00425-026-04941-z

**Published:** 2026-02-03

**Authors:** Annelotte van Dieren, Andras Bittner, Bernhard Wurzinger, Leila Afjehi‑Sadat, Wolfram Weckwerth, Markus Teige, Ute C. Vothknecht

**Affiliations:** 1 Institute for Cellular and Molecular Botany, University of Bonn, Kirschallee 1, 53115 Bonn, Germany; 2 Molecular Systems Biology (MOSYS), Department of Functional and Evolutionary Ecology, University of Vienna, Djerassiplatz 1, 1030 Vienna, Austria; 3 Mass Spectrometry Unit, Research Support Facilities, University of Vienna, Djerassiplatz 1, 1030 Vienna, Austria; 4 Institute of Plant Biotechnology and Cell Biology, Department of Biotechnology and Food Sciences, BOKU University, Vienna, Austria; 5 Vienna Metabolomics Center (VIME), University of Vienna, Djerassiplatz 1, 1030 Vienna, Austria

**Correction: Planta (2026) 263:23**

10.1007/s00425-025-04891-y

In this article, the dataset identifier in ‘Data Availability’ statement was incorrectly given as PXD069039. The corrected dataset identifier is given below.

**Data availability** The authors upon reasonable request can provide the data presented in this study. The MS data have been deposited to the ProteomeXchange Consortium via the PRIDE (Perez-Riverol et al. 2025) partner repository with the dataset identifier PXD069030.

