# Supplementary material for: With or without a Ca2+ signal?: a proteomics approach toward Ca2+-dependent and -independent changes in response to oxidative stress in Arabidopsis thaliana
Source: Planta. 2025 Dec 5;263(1):23. doi: 10.1007/s00425-025-04891-y (PMC12680889; doi:10.1007/s00425-025-04891-y)
Supplement: Supplementary file 1 — Supplementary file1 (PDF 234 KB) [file 425_2025_4891_MOESM1_ESM.pdf]

With or without a Ca^{2+} signal?
A proteomics approach towards Ca^{2+} dependent and independent proteome changes in response to oxidative stress in *A. thaliana*

Annelotte van Dieren, Andras Bittner, Bernhard Wurzinger, Leila Afjehi-Sadat, Wolfram Weckwerth, Markus Teige and Ute C. Vothknecht

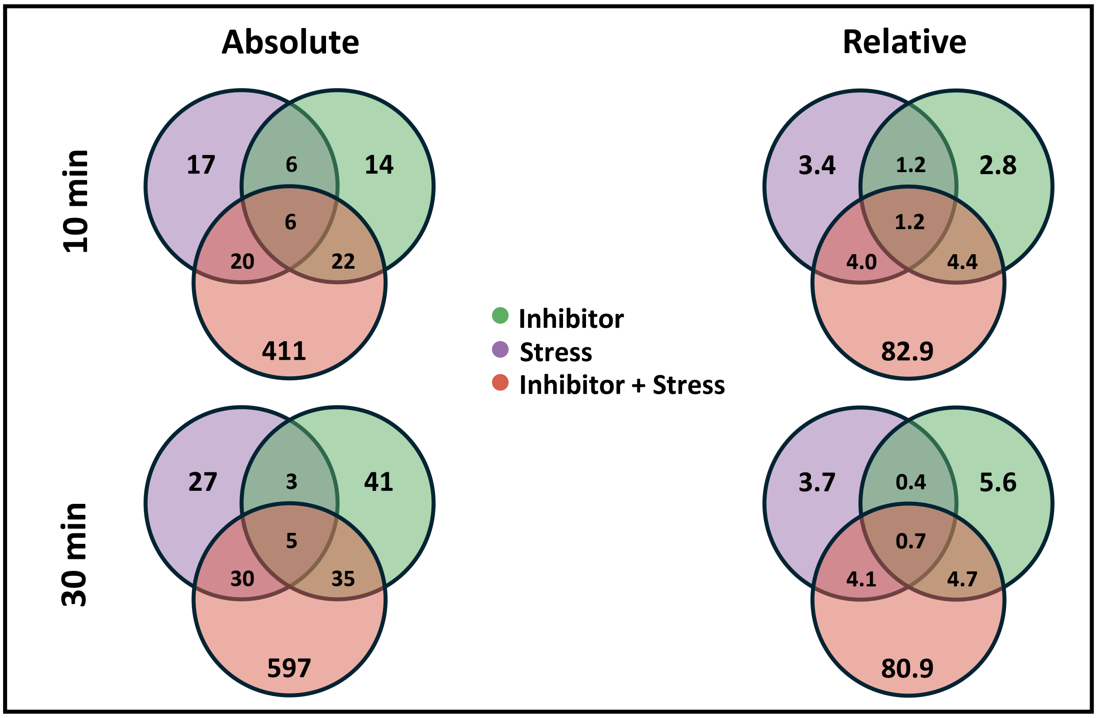

Supplementary Fig. S1: Absolute and relative (% of total identified DAPs) number of DAPs for 10 min (top) and 30 min (bottom) of stress treatment.

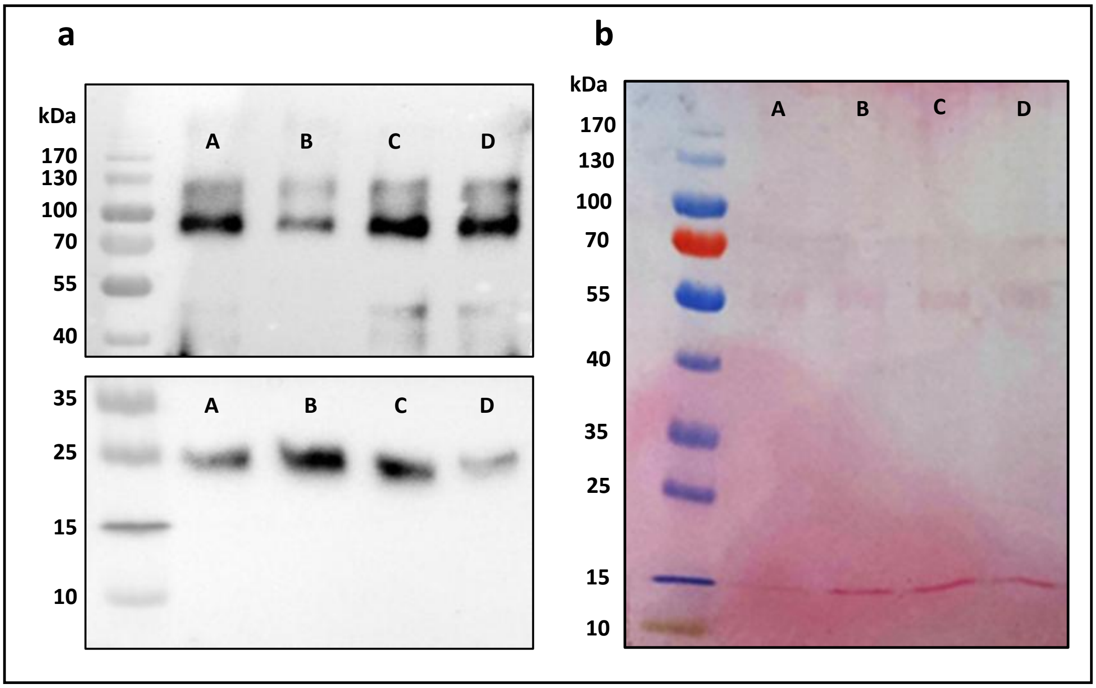

Supplementary Fig. S2: a: Comparison of abundance of PAL (top blot) and TIP (bottom blot) in an aliquot of the leaf samples that were used for proteomics analysis. The abundance of PAL and TIP among the different treatments with a duration of 10 minutes (A= Control, B= Stress, C= Inhibitor, D= Inhibitor+Stress) was determined by immunodetection using a specific antibody against PAL and TIP. The Thermo Scientific™ PageRuler prestained™ protein ladder was used to indicate the size of the detected proteins. Due to the distinct sizes of the two proteins of interest the same membrane was used and cut in two before incubation with the primary antibodies. **b**: Ponceau staining of the membrane before incubation with the primary antibodies

Supplementary Table S1: Analysis of GO functional categories for the H_2O_2 responsive proteins identified after 10 min of treatment.

	Pathway database (GO)	GO Functional category	# Proteins	Proteins
Strict Ca^{2+} dependent	Biological process	Cellular response to stimulus	6	RPT2 DJ1A GRXC5 UBA2 CAM6 ADF4
	Biological process	Response to stress	6	DJ1A TDX PAL2 GRXC5 UBA2 ADF4
	Biological process	Response to abiotic stimulus	4	RPT2 TDX PAL2
	Biological process	Catabolic process	3	DJ1A PAL2 UBA2
	Biological process	Cellular component organization	3	TDX GRXC5 ADF4
	Biological process	Signaling	3	RPT2 CAM6 ADF4
	Molecular function	Oxidoreductase activity	3	APR2 TDX GRXC5
	Molecular function	Transferase activity	2	STR2 UBA2
	Molecular function	Lyase activity	2	DJ1A PAL2
Partial Ca^{2+} dependent	Biological process	Response to stress	4	CRSP MLP43 THF1 SGT1B
	Biological process	Developmental process	3	CRSP RPS9M SGT1B
	Biological process	Catabolic process	3	THF1 SGT1B ECI3
	Biological process	Response to abiotic stimulus	3	THF1 SGT1B ECI3
	Biological process	Response to chemical	3	CRSP THF1 SGT1B
	Biological process	Anatomical structure development	3	CRSP RPS9M SGT1B
	Biological process	Cellular component organization	3	WLIM1 THF1 PRMT11
	Molecular function	Transferase activity	2	LSMT-L PRMT11
	Molecular function	Hydrolase activity	2	PAP1 CRSP
Ca^{2+} independent	Biological process	Response to stress	3	DGK5 LOX2 PP7
	Biological process	Cellular response to stimulus	2	DGK5 PP7
	Biological process	Signaling	2	DGK5 PP7
	Biological process	Response to external stimulus	2	LOX2 PP7

Supplementary Table S2: Analysis of GO functional categories for the H_2O_2 responsive proteins identified after 30 min of treatment.

	Pathway database (GO)	GO Functional category	# Proteins	Proteins
Strict Ca^{2+} dependent	Biological process	Multicellular organismal process	6	ATKDSA2 STR2 CINV1 FIP37 SCE1 FER1
	Biological process	Developmental process	6	ATKDSA2 STR2 CINV1 FIP37 SCE1 FER1
	Biological process	Cellular component organization or biogenesis	6	WLIM1 ATKDSA2 RPL34A CINV1 XRN3 CSP1
	Biological process	Response to stress	6	CINV1 PDF2.2 CPK3 CSP1 FER1 CSD3
	Biological process	Response to chemical	6	CINV1 SCE1 CPK3 CSP1 FER1 CSD3
	Biological process	Anatomical structure development	6	ATKDSA2 STR2 CINV1 FIP37 SCE1 FER1
	Biological process	Cellular component biogenesis	5	WLIM1 ATKDSA2 RPL34A CINV1 XRN3
	Biological process	Reproduction	4	ATKDSA2 STR2 SCE1 FER1
	Biological process	Reproductive process	4	ATKDSA2 STR2 SCE1 FER1
	Biological process	Developmental process involved in reproduction	4	ATKDSA2 STR2 SCE1 FER1
	Biological process	Response to abiotic stimulus	4	CPK3 CSP1 FER1 CSD3
	Biological process	Cellular component organization	4	WLIM1 ATKDSA2 CINV1 CSP1
	Biological process	Localization	3	CPK3 FER1 VPS26A
	Molecular function	Oxidoreductase activity	5	SCE1 FER1 CSD3 PSBD
	Molecular function	Transferase activity	4	ATKDSA2 STR2 SCE1 CPK3
	Molecular function	Hydrolase activity	2	CINV1 XRN3
	Molecular function	Lyase activity	2	PDX12 ASB2
	Molecular function	Small molecule binding	2	SCE1 CPK3
Partial Ca^{2+} dependent	Biological process	Cellular component organization	5	FREE1 RPL6B CSP3 PME44 GRIP
	Biological process	Localization	4	FREE1 NUP50A ATPC GRIP
	Biological process	Response to stress	4	MLP43 CSP3 MES10 PME44
	Biological process	Response to abiotic stimulus	3	FD2 CSP3 MES10
	Biological process	Response to external stimulus	2	PME44 ATPHOS32
	Biological process	Response to chemical	2	MES10 ATPHOS32
	Biological process	Hydrolase activity	4	ZFN1 MES10 PME44 ATPHOS32
	Molecular function	Small molecule binding	3	2NADP-ME4 ATPHOS32
	Molecular function	Structural molecule activity	2	RPL6B RPL8A
Ca^{2+} Indep.	Molecular function	Oxidoreductase activity	4	LOX2 CYP97C1 PPOX1 RBCS-1B
